# Unmasking the intrinsic mechanistic limits of manganese Prussian blue analogues in aqueous Zn-ion batteries

**DOI:** 10.1039/d6sc02408d

**Published:** 2026-05-20

**Authors:** Sankhadip Saha, Yuan Shang, Ruichen Ma, Xiaoran Zheng, Priyank Kumar, Dipan Kundu

**Affiliations:** a LBRI, School of Chemical Engineering, UNSW Sydney Kensington NSW 2052 Australia yuan.shang@unsw.edu.au d.kundu@unsw.edu.au; b School of Chemical Engineering, UNSW Sydney Kensington NSW 2052 Australia priyank.kumar@unsw.edu.au; c School of Chemistry, UNSW Sydney Kensington NSW 2052 Australia

## Abstract

Manganese Prussian blue analogues (Mn-PBAs) are widely investigated as cathodes for aqueous zinc-ion batteries (AZIBs) owing to their open framework and potential two-electron redox, yet their true electrochemical stability and charge-storage mechanism remain contentious. Here, we systematically disentangle the roles of electrolyte composition and compositional variations in Mn-PBAs using a combination of electrochemical analysis, *operando* X-ray diffraction, solution-phase analysis, microscopy, soft X-ray absorption spectroscopy, and density functional theory. It is revealed that, independent of composition and electrolyte formulation, Mn-PBAs do not operate as stable intercalation hosts with aqueous zinc-ion electrolytes. Initial Na^+^ deintercalation triggers a monoclinic-to-cubic transition, accompanied by Mn^3+^-driven disproportionation and manganese dissolution, followed by electrochemical deposition of manganese oxide that contributes capacity beyond one electron, reaching 136 mA h g^−1^ in the Na^+^–Zn^2+^ dual salt electrolyte compared to 111 mA h g^−1^ in the Zn^2+^ based single salt system after activation. Subsequent discharge induces irreversible conversion of the Mn-PBA framework to a rhombohedral Zn-PBA phase, with later cycling dominated by the solid-solution behavior of Zn-PBA alongside repeated manganese dissolution–redeposition. As a result, the capacity of Mn-PBA converges toward that of Zn-PBA and decays rapidly, with only 60% (dual-salt) and 43% (single-salt) retention after 100 cycles. While the dual-salt electrolyte delays manganese dissolution and enables partial (de)intercalation within Zn-PBA, it does not alter the fundamental reaction pathway. Across the compositions investigated, differences are reflected primarily in activation behavior and electrochemical kinetics. These findings reconcile reports of high-rate cycling stability at modest capacities and establish intrinsic limitations of Mn-PBAs in AZIBs, highlighting the need for interfacial and electrolyte strategies to suppress Mn^3+^ disproportionation.

## Abstract 2

Mn-PBAs in aqueous Zn-ion batteries do not act as stable intercalation hosts but follow a dissolution–redeposition-driven conversion pathway, leading to Zn-PBA and manganese oxide phases that dominate electrochemical behavior and limit stability.

## Graphical abstract

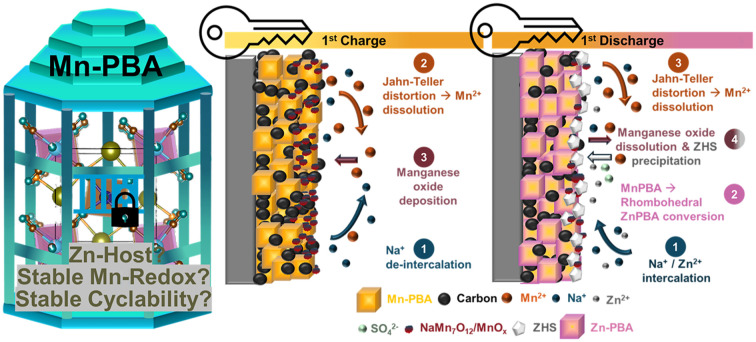

## Introduction

Aqueous batteries, built on water-based electrolytes, have emerged as a compelling alternative to conventional nonaqueous Li-ion systems, offering inherent safety, cost-effectiveness, and environmental compatibility. These attributes make them particularly attractive for stationary energy storage, where volumetric or gravimetric energy densities are less critical than affordability and operational safety. The search for suitable chemistries has spanned both monovalent (Li^+^/Na^+^/K^+^) and multivalent ions (Zn^2+^/Mg^2+^/Al^+3^/Ca^2+^),^1,2^ each bringing unique opportunities and challenges. Among these, zinc-ion systems have garnered tremendous attention, primarily due to the high electrochemical reversibility of the zinc metal anode (∼0.76 V *vs.* SHE) in aqueous electrolytes – a distinct advantage over other chemistries that often rely on capacitive carbon-based anodes with inherently low capacity.

Yet zinc is far from a perfect solution. Despite its promise, the technology faces persistent hurdles: dendritic growth and hydrogen evolution-driven corrosion remain formidable barriers to long-term cycling under practical conditions. Over the past five years, intensive research efforts have shifted zinc-ion batteries from a niche curiosity to a serious contender for grid-scale storage, with advances in electrolyte engineering, interface stabilization, and cathode design.^3–8^ However, as with other multivalent-ion systems, cathode development continues to be the Achilles' heel. The palette of viable positive electrodes is rather narrow, dominated by MnO_2_ polymorphs,^5,9,10^ vanadium oxide bronzes,^3,11–13^ Prussian blue analogues (PBAs),^6,14–16^ and organic frameworks,^17–19^ and each is plagued by stability woes and limited capacity under practically relevant conditions. While MnO_2_ and vanadium-based cathodes have been extensively dissected, PBAs remain comparatively underexplored despite their structural versatility and theoretical promise.

PBAs, or metal hexacyanometallates, with the general formula A_*x*_M_1_[M_2_(CN)_6_]·*n*H_2_O (A = mobile cation; M_1_, M_2_ = transition metals), have emerged as versatile candidates for AZIBs.^20^ Their open three-dimensional framework, large interstitial voids, and tunable redox properties enable efficient insertion of both monovalent and multivalent ions such as K^+^, Na^+^, Zn^2+^, Al^3+^, and Mg^2+^, positioning PBAs as promising materials for next-generation energy storage.^21–29^ Within this family, manganese-based PBAs (Mn-PBAs; where M_1_: Mn and M_2_: Fe) stand out for their high theoretical capacity and output voltage, driven by the synergistic redox activity of Mn and Fe.^30–33^ The use of Mn-PBA as a cathode in AZIBs was first demonstrated by Hou *et al.*^34^ employing a 1 M Na_2_SO_4_–1 M ZnSO_4_ dual salt electrolyte with sodium dodecyl sulphate as an electrolyte additive, showcasing a capacity of 140 mA h g^−1^ with 74% retention after 2000 cycles at a 5C rate (1C = 160 mA g^−1^). Subsequent studies have also investigated its performance in single-salt ZnSO_4_ electrolytes^30,31^ and various electrolyte additives have been explored to suppress degradation and improve cycling stability.^33,35^

Despite significant efforts, the long-term stability of Mn-PBAs in AZIBs while ensuring more than one-electron redox utilization remains hindered by intrinsically poor electrical conductivity and pronounced structural instability arising from Mn^3+^ induced Jahn–Teller distortions and irreversible Zn^2+^ intercalation, which leads to the formation of Zn-PBA phases with MnO_2_ like phase formation.^36^ Most reports demonstrate extended cycling at high rates, typically accompanied by modest specific capacities. Attempts to access deeper charge storage or multi-electron utilization at moderate rates often trigger rapid capacity decay, yet the mechanistic origins, particularly the interplay between phase evolution and electrolyte composition, remain poorly understood.^37^ High salt concentrations with dual-ion environments, frequently employed to suppress water activity, may inadvertently influence interfacial chemistry and phase transitions, but these effects are rarely isolated or quantified.^38^

Furthermore, the identity of the electrochemically active phase during prolonged cycling remains insufficiently resolved. In particular, the frequent observation of stable cycling at high current densities has often been interpreted as evidence for stabilized Mn-PBA redox, even though such conditions typically access only a limited fraction of the theoretical capacity. Without direct insight into phase evolution and redox chemistry, it remains unclear whether the reported stability reflects reversible charge storage within the Mn-PBA framework or the emergence of alternative, kinetically favoured charge-storage phases under electrochemical cycling. Resolving this distinction is essential for correctly interpreting electrochemical performance metrics and for identifying meaningful design strategies for durable Mn-PBA cathodes in AZIBs.

Here, we address these unresolved questions by systematically probing sodium manganese Prussian blue analogues in aqueous Zn-ion batteries using both single-salt (ZnSO_4_) and dual-salt (Na_2_SO_4_ + ZnSO_4_) electrolytes, while systematically varying the composition of the Mn-PBA host lattice. By combining electrochemical measurements with *operando* X-ray diffraction, solution-phase analysis, electron microscopy, X-ray absorption spectroscopy, and density functional theory calculations, we track phase evolution, interfacial chemistry, and redox activity in real time and as a function of cycling. This integrated approach allows us to disentangle the roles of electrolyte composition and material compositions, identify the dominant charge-storage phases under different cycling conditions, and clarify the mechanistic origins of apparent stability and capacity in Mn-PBA cathodes.

## Results and discussion

### Structural and morphological insights into the synthesized Mn-PBAs

**Fig. 1:**
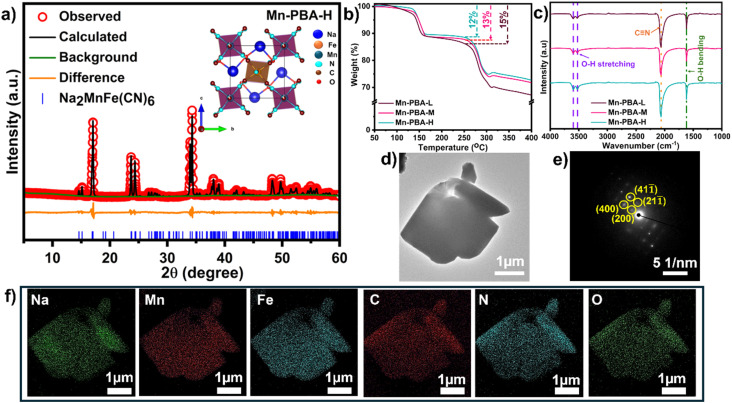
Integrated structural, thermal, and morphological characterization of Mn-PBA samples. (a) Rietveld refinement (*R*_wp_: 3.08%, *χ*^2^(gof): 3.31) of the powder XRD data of as-prepared Mn-PBA-H, using ICSD reference #266392, confirms a monoclinic (*P*2_1_/*n*) phase. Observed data points (red ‘o’ symbol); calculated profile (black line); difference profile (orange line); Bragg positions (blue ticks). (b) TGA showing stepwise mass loss associated with the removal of adsorbed and interstitial water, demonstrating higher relative thermal stability of Mn-PBA-H compared to Mn-PBA-L. (c) FT-IR spectrum revealing O–H bending and stretching signatures, along with the characteristic C≡N stretching band. (d) A representative STEM image showing the deformed cuboid-like morphology of Mn-PBA-H particles. (e) SAED pattern displaying sharp diffraction spots consistent with the monoclinic lattice. (f) STEM-EDS elemental mapping of the particle showing uniform distribution of Na, Mn, Fe, C, and N throughout.

To systematically examine the influence of compositional variations, including sodium content, vacancy concentration, and lattice water, on the structural and electrochemical behavior of Mn-PBAs, three distinct compositions, Mn-PBA-H (high Na – Na_1.96_Mn[Fe(CN)_6_]·2.37H_2_O), Mn-PBA-M (medium Na – Na_1.79_Mn[Fe(CN)_6_]_0.93_·2.67H_2_O), and Mn-PBA-L (low Na – Na_1.35_Mn[Fe(CN)_6_]_0.83_·3.06H_2_O) (Table S1), were synthesized by varying NaCl concentration during co-precipitation (see Experimental details). Following synthesis, thorough washing removes any residual NaCl, ensuring that the measured elemental composition reflects intrinsic framework chemistry. It is important to note that NaCl is employed here as a synthesis regulator to systematically tune sodium incorporation into the Mn-PBA framework, rather than as an independent compositional variable for electrochemical comparison. Rietveld refinement of the powder X-ray diffraction (XRD) data confirmed that all three variants crystallize in the monoclinic *P*2_1_/*n* phase, consistent with the Na_2_MnFe(CN)_6_ framework^39^ (Fig. 1a and S1–2). Among these, Mn-PBA-H exhibits sharp, well-defined diffraction peaks, reflecting high crystallinity and lower structural disorder, as well as the absence of or minimal Mn and [Fe(CN_6_)]^4−^ deficiencies (Fig. 1a).

Water molecules in PBAs exist in three forms: adsorbed, interstitial, and coordinated, and this is evident from the thermogravimetric analysis (TGA), which reveals a stepwise mass loss corresponding to these different water species (Fig. 1b). Upon heating, adsorbed water is released first (below 180 °C), followed by interstitial water confined within the framework (between 180–280 °C), and finally the coordinated water (above 280 °C), which is chemically bound at the transition metal sites, particularly at the Mn vacancies. Incorporating sodium-EDTA as a chelating agent during the synthesis effectively reduces the adsorbed water content; however, the slower nucleation kinetics favor the retention of water within the lattice.^39^ Mn-PBA-H displays a gradual degradation profile, whereas Mn-PBA-L undergoes rather steep mass loss beyond 280 °C, reflecting its higher defect density and weaker lattice integrity. Fourier-transform infrared (FTIR) spectra (Fig. 1c) further confirm the presence of water through characteristic O–H bending (1620 cm^−1^) and stretching (3520 cm^−1^) vibrations, alongside strong C≡N stretching at 2062 cm^−1^, consistent with the structural and functional features of the PBA framework.

Morphological analysis using scanning electron microscopy (SEM) and energy-dispersive X-ray spectroscopy (EDS) mapping reveals distorted cuboid-like particles with dimensions ranging from 2 to 10 µm across all sodium variants (Fig. S3–S5). Here, the inclusion of Na-EDTA (EDTA: ethylenediaminetetraacetic acid) as a chelating agent slows the nucleation kinetics, enabling the formation of larger crystallites. When used in conjunction with elevated sodium chloride concentrations, this approach promotes greater sodium incorporation into the PBA lattice, presenting a simple yet powerful method for precise composition control. Scanning transmission electron microscopy (STEM) imaging of the Mn-PBA-H particles, shown in Fig. 1d, confirms the deformed cuboid-like morphology, while selected-area electron diffraction (SAED) analysis (Fig. 1e) on the particle reveals sharp diffraction spots consistent with the monoclinic lattice. STEM-EDS elemental mapping of Na, Mn, Fe, C, and N on the particle corroborates the uniformity of elemental distribution. While minor variations in surface area cannot be completely excluded, all samples are designed to exhibit comparable morphology and particle size, ensuring that differences in electrochemical behavior primarily arise from electrolyte chemistry and compositional effects rather than morphological factors.

### Dissecting electrolyte-dependent mechanisms in charge storage

**Fig. 2:**
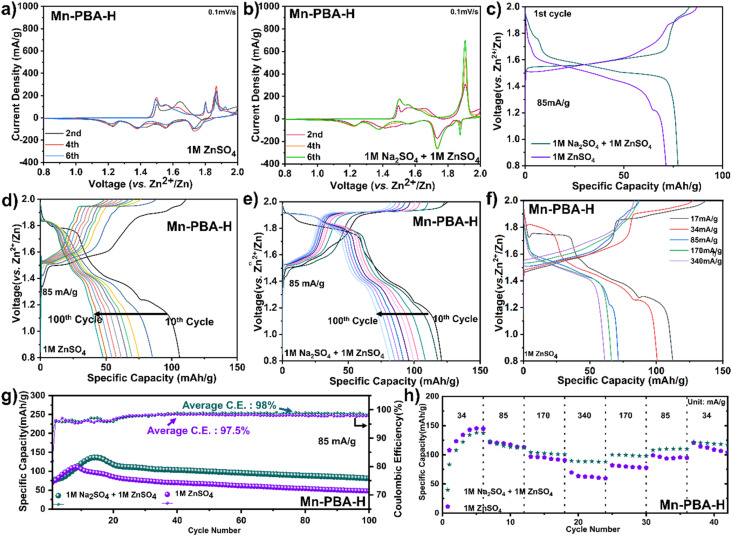
Electrochemical performance of Mn-PBA-H in the two electrolytes. (a) CV curves recorded at 0.1 mV s^−1^ for Mn-PBA-H in 1 M ZnSO_4_, and (b) in 1 M ZnSO_4_ + 1 M Na_2_SO_4_. (c) Initial capacity–voltage profiles (first cycle) for both electrolytes at 85 mA g^−1^. (d) Capacity–voltage profiles over 10–100 cycles showing evolution of the redox plateaus in 1 M ZnSO_4,_ and (e) in 1 M ZnSO_4_ + 1 M Na_2_SO_4_. (f) Charge–discharge voltage profiles at different current densities in 1 M ZnSO_4_. (g) Long-term cycling stability of Mn-PBA-H in both electrolytes. (h) Rate-capability comparison of Mn-PBA-H between the two electrolytes.

Cyclic voltammetry (CV) profiles (Fig. 2a and b) for Mn-PBA-H in 1 M ZnSO_4_ at 0.1 mV s^−1^ show three anodic or oxidation peaks at ∼1.50 V, 1.80 V, and 1.87 V, along with a couple of broad features between ∼1.50 and 1.70 V. The corresponding cathodic or reduction scan exhibits peaks at 1.73 V, 1.55 V, 1.38 V, and 1.22 V. In the dual-salt electrolyte (1 M Na_2_SO_4_ + 1 M ZnSO_4_), two dominant anodic peaks around 1.50 V and 1.90 V and four cathodic peaks at 1.87 V, 1.74 V, 1.34 V, and 1.22 V are observed. While the anodic peak near 1.50 V shows similar intensity in both systems, the peak at ∼1.90 V is significantly more pronounced in the dual-salt electrolyte, indicating enhanced electrochemical activity at the high voltage.

The initial charge–discharge cycle at 85 mA g^−1^ (Fig. 2c) does not yield the highest capacity, but the dual-salt electrolyte demonstrates better reversibility than the single-salt 1 M ZnSO_4_. At this current, which is equivalent to a 0.5C rate, both systems require several activation cycles to reach peak capacities – 136 mA h g^−1^ for the dual-salt electrolyte and 111 mA h g^−1^ for the single-salt. However, rapid capacity fading is observed, with retention of 60% (dual-salt) and 43% (pure Zn^2+^) after 100 cycles (Fig. 2d–g). This trend is evident in the voltage–capacity profiles as well (Fig. 2d and e), which show progressive loss of the redox plateaus. Rate capability data (Fig. 2f and h) further highlight kinetic limitations in pure ZnSO_4_ electrolyte, where capacity declines sharply with increasing current density, while the dual-salt system maintains relatively stable and higher capacity across rates. These observations highlight clear differences between the two electrolyte systems and point to unresolved aspects concerning the origin of rapid capacity fading as well as the role of electrolyte environment in governing structural evolution and stability. Additional measurements in higher-concentration ZnSO_4_ electrolytes (2 M and 3 M) show that increasing ionic strength alone does not reproduce the electrochemical behavior observed in the dual-salt system (Fig. S6), indicating that the differences arise from the coupled influence of electrolyte environment and cation composition. To probe the underlying phase evolution and reaction mechanism, *operando* XRD and complementary physicochemical analyses were employed.

**Fig. 3:**
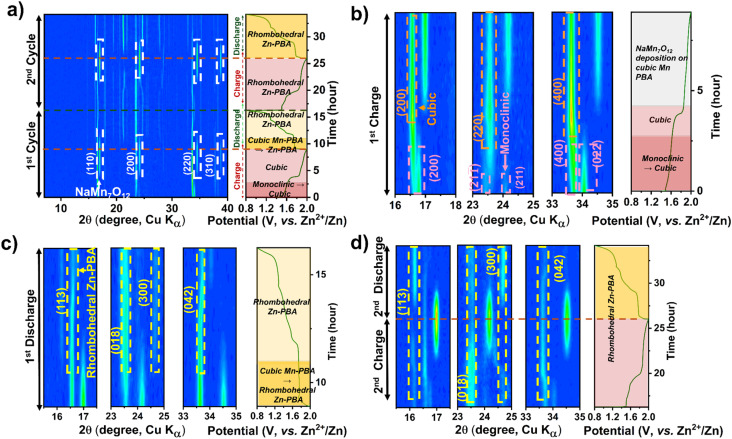
*Operando* XRD contour plots showing the phase evolution of Mn-PBA-H in (a–d) 1 M ZnSO_4_ electrolyte. Panels (a) show the first two charge–discharge cycles, (b) the first charge, (c) the first discharge, and (d) the second charge–discharge cycle. Key phase transformations and structural evolutions during cycling are highlighted by arrows, labels, and legends.

*Operando* XRD data collected during cell cycling, shown in Fig. 3a–d for Mn-PBA-H in the 1 M ZnSO_4_ electrolyte, reveal the phase evolution during the initial two charge–discharge cycles. Firstly, the monoclinic Mn-PBA-H phase transforms to a cubic phase at the onset of charging, around 1.6 V, followed by the emergence of pronounced diffraction peaks corresponding to sodium manganese oxide – indexed to (110), (200), (220), and (310) planes of NaMn_7_O_12_ (ICSD #19022) – as the potential rises to 1.8 V. The phase transition from the monoclinic to cubic during the first charge is further highlighted in Fig. 3b. The characteristic diffraction peaks of the monoclinic phase, namely the (200), (211), (211̄) and (400), gradually give way to form the (200) and (400) peaks characteristic of the cubic phase. Intriguingly, during the discharge, alongside the dissolution of the manganese oxide phase, the cubic Mn-PBA Na_*x*_MnFe(CN)_6_ undergoes an irreversible transformation to rhombohedral Zn-PBA Na_2_Zn_3_[Fe(CN)_6_]_2_, as evident from the emergence of its characteristic diffraction peaks (Fig. 3c). Holding the cell at the open-circuit potential upon charging (1st) and tracking the XRD evolution confirmed the stability of the charged cubic phase and the deposited sodium manganese oxide phase (Fig. S7), with no signs of dissolution. This observation indicates that the subsequent transformation to Zn-PBA during discharge requires applying a reduction current – suggesting that it is electrochemically driven – and does not proceed measurably at OCV on the timescale of our experiment. During the second charge, the rhombohedral Zn-PBA persists, exhibiting only a solid-solution type transition while the manganese oxide phase reappears (Fig. 3d). Since the specific capacity of Mn-PBA-H in 1 M ZnSO_4_ peaks around the fifth cycle at 17 mA g^−1^ (0.1C), the XRD structural evolution was probed *operando* up to this point. At this point, the phase evolution involves a solid-solution transition of rhombohedral Zn-PBA during discharge/charge, accompanied by dissolution and redeposition of the manganese phase (Fig. S8).

**Fig. 4:**
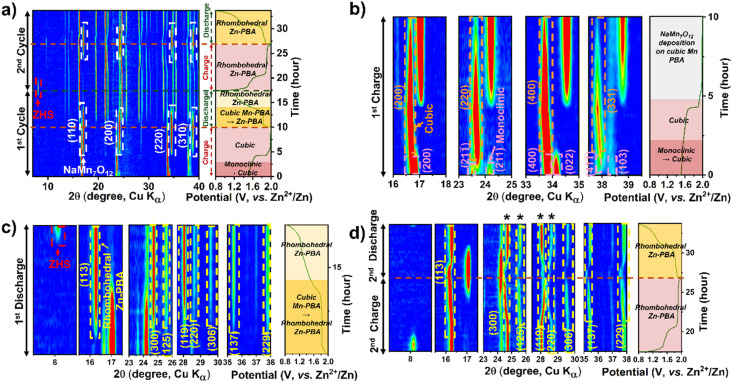
*Operando* XRD contour plots showing the phase evolution of Mn-PBA-H in 1 M Na_2_SO_4_ + 1 M ZnSO_4_ electrolyte. Panel (a) shows the first two charge–discharge cycles, (b) the first charge, (c) the first discharge, and (d) the second charge–discharge cycle. Key phase transformations and structural evolutions during cycling are highlighted by arrows, labels, and legends.

A similar electrochemical transformation is observed with the dual-ion 1 M Na_2_SO_4_ + 1 M ZnSO_4_ electrolyte system (Fig. 4a–d). At the onset of the first charge cycle, the monoclinic phase transforms to the cubic Mn-PBA phase, as observed in ZnSO_4_. The deposition of sodium manganese oxide above 1.8 V is evident from the emergence of prominent diffraction peaks (Fig. 4a and b) of NaMn_7_O_12_. Unlike in the ZnSO_4_ electrolyte, where ZHS (Zn_4_SO_4_(OH)_6_·nH_2_O) formation^40^ is not evident in the *operando* XRD profile, here, a pronounced diffraction peak corresponding to the (001) plane of ZHS appears near the end of discharge (Fig. 4a and c). The ZHS forms *via* the reaction of OH^−^ with Zn^2+^ and SO_4_^2−^ ions in the electrolyte, triggered by the consumption of H^+^ during sodium manganese oxide dissolution on discharge. During the subsequent charging step (Fig. 4d), the release of H^+^ back into the electrolyte, coupled with the deposition of the sodium manganese oxide phase, leads to the dissolution of ZHS, causing its disappearance. Consistent with the observation in the ZnSO_4_ electrolyte, the growth of the rhombohedral Zn-PBA phase is observed below 1.8 V during the first discharge.

Interestingly, unlike in the pure Zn^2+^ electrolyte, a gradual transformation of cubic Mn-PBA to rhombohedral Zn-PBA is also observed in the dual-ion electrolyte when the *operando* cell is held up at OCV for over 10 h following the first charge (Fig. S9), marking the first major distinction between the two electrolytes. No dissolution of the deposited oxide phase is detected during the rest period, however. In the fifth cycle, cathode evolution is dominated by the solid-solution transition of rhombohedral Zn-PBA throughout charge–discharge. Unlike in the single-salt system, diffraction peaks corresponding to the manganese oxide become much weaker in the fifth cycle, which may be attributed to its transition to an amorphous state over repeated cycling, rendering it undetectable by XRD. Whereas the characteristic (113) and (300) diffraction peaks of the rhombohedral phase display a small shift in peak positions between discharge and charge (Fig. S10), indicating a change in the interplanar *d*-spacing, most plausibly associated with ion (de)intercalation in Zn-PBA. Notably, this intercalation signature is not detected in the 1 M ZnSO_4_ system. Collectively, these observations suggest that Na^+^ availability and increased ionic strength modify interfacial equilibria, rebalancing dissolution–redeposition processes *versus* framework-based intercalation.

**Fig. 5:**
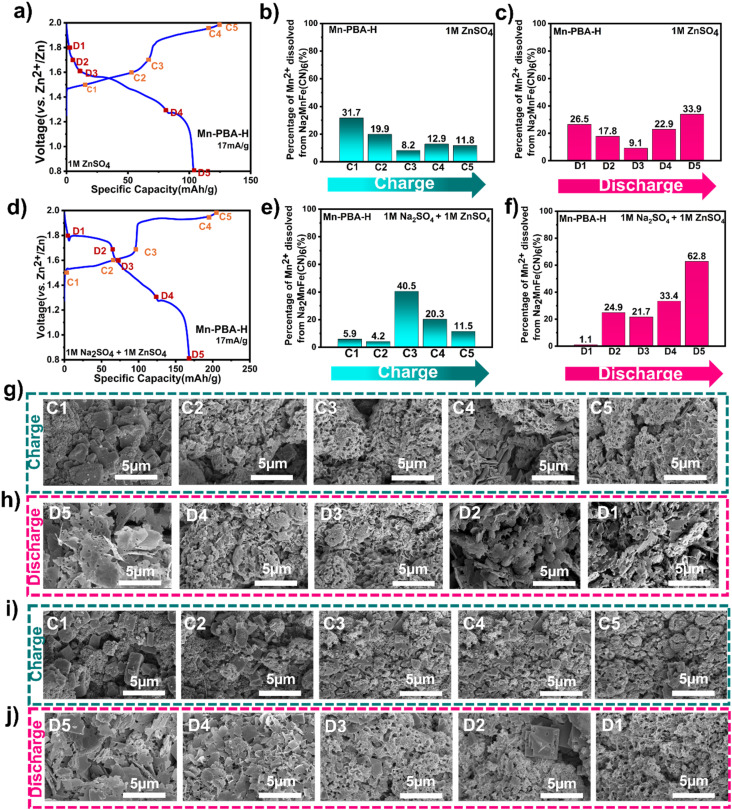
Compositional and morphological evolution of Mn-PBA-H during the first charge–discharge cycle in the two electrolytes. (a–c, g and h) for 1 M ZnSO_4_ and (d–f, i and j) for 1 M Na_2_SO_4_ – 1 M ZnSO_4_ electrolytes. (a and d) Voltage profile of the first charge–discharge cycle, with electrodes selected at the labeld states of charge during charging (C1–C5) and discharging (D1–D5) for analysis; (b, c, e and f) evolution of Mn^2+^ concentration in the electrolyte at different states of charge, quantified by ICP-OES, indicating manganese dissolution and redeposition, respectively; (g, h, i and j) representative SEM images showing electrode morphological evolution, including the deposition of nanorod-like sodium manganese oxide during charge and its subsequent dissolution, accompanied by the formation of plate-like ZHS during discharge.

ICP-OES analysis (Fig. 5a–f) during the first charge–discharge reveals slightly different manganese dissolution behavior between the two electrolytes at lower potentials. The cells were stopped and disassembled at the designated points on the polarization profile – at the same potentials for the two electrolytes – as shown in Fig. 5a and d, and the electrolyte extracted from the separator was analysed by ICP-OES to quantify dissolved Mn^2+^. In ZnSO_4_ (Fig. 5b), the highest amount of Mn^2+^ is found very early in the charge (C1/1.5 V), but then decreases until C3/1.7 V due to manganese oxide deposition before increasing again slightly during the second half of the charging (C4/1.9 V and C5/2 V).

In comparison, the Mn^2+^ dissolution is initially minor in the dual-ion electrolyte (Fig. 5e), even when a significant charge capacity (C2/1.6 V) has been delivered. The Mn^2+^ concentration in the electrolyte peaks halfway through the charge, at around 1.7 V (C3), before declining again as charging progresses due to deposition of Mn^2+^ as the solid manganese oxide phase. The difference between the two electrolytes most plausibly arises from the altered electrolyte environment, including ionic strength and cation composition, suppressing Jahn–Teller distortion mediated disproportionation of Mn^3+^ at the electrode–electrolyte interface during the early stages of charging. Since both electrolytes were adjusted to pH 4.5, differences arising from pH can be excluded. Regardless, the Mn^2+^ dissolution during charging in both electrolytes can be linked to the Na^+^ de-intercalation and accompanying disproportionation of oxidized Mn (Mn^3+^) in the lattice at the Mn-PBA particle surface and transformation of monoclinic Mn-PBA into cubic Mn-PBA. Cubic Mn-PBA to rhombohedral Zn-PBA transformation during discharge plausibly follows a similar Mn^3+^ disproportionation-dissolution path accompanying Zn^2+^ intercalation (in 1 M ZnSO_4_) or Na^+^/Zn^2+^ (in 1 M Na_2_SO_4_–1 M ZnSO_4_) co-intercalation. However, analysis of soluble Mn^2+^ in the electrolyte during discharge (Fig. 5a, c, d and f) reveals a noticeable difference between the two electrolytes. In the single-salt system, the disproportionation-dissolution is more aggressive (Fig. 4c), leading to an increase in Mn^2+^ in the electrolyte early in the discharge. The Mn^2+^ concentration decreases marginally as the voltage reaches 1.6 V (the reason remains unclear), then increases again with further discharge, owing to electrochemical dissolution of the manganese oxide phase. In the dual-salt electrolyte, initial Mn^2+^ dissolution is small, and its concentration in the electrolyte rises progressively with discharge.

SEM imaging of the morphological evolution (Fig. 5g–j and S11) during charge and discharge confirms nano-rod-like sodium manganese oxide deposition during charging, while plate-like ZHS becomes dominant on the electrode surface at the end of discharge. The morphology evolution – dictated by the manganese oxide phase deposition during charge and its dissolution, accompanied by ZHS deposition during discharge – becomes consistent over cycling. Thus, overall, while both electrolytes exhibit broadly similar phase transition behaviour – monoclinic → cubic → rhombohedral Zn-PBA – coupled with manganese dissolution–redeposition and ZHS formation, the dual-ion system offers marginal mitigation by delaying Mn dissolution and enabling partial ion intercalation into the Zn-PBA framework. In this regard, to assess whether Zn-PBA-like behavior emerges as the dominant electrochemical response during cycling, Zn-PBA was chemically synthesized (Fig. S12), and the galvanostatic performance of the corresponding electrode in the two electrolytes was compared with that of Mn-PBA-H. As shown in Fig. S13–S14, in the 1 M ZnSO_4_ electrolyte, Zn-PBA shows rapid degradation, with the capacity dropping to ∼10 mA h g^−1^ within 30 cycles. In contrast, in the dual-salt electrolyte, Zn-PBA demonstrates a much-improved stability with a notable capacity corresponding to Na^+^ (de)intercalation. Within 50 cycles, in both electrolytes, the capacity of Mn-PBA-H drops to that of the pure Zn-PBA. It should be noted that the electrochemically formed phase is part of a dynamically evolving composite that includes Zn-PBA together with redeposited manganese oxide species, and therefore is not directly equivalent to the chemically synthesized Zn-PBA.

### Establishing the role of compositional variations in electrochemical behavior

Evaluation of the high-sodium composition (Mn-PBA-H) in both electrolyte systems has revealed that the dual-salt electrolyte exhibits greater stability than its Zn^2+^ containing single-salt counterpart. To further examine Mn-PBA compositional effects on the observed electrochemical behavior, medium (Mn-PBA-M) and low-sodium (Mn-PBA-L) variants were subsequently studied in the dual-salt electrolyte.

**Fig. 6:**
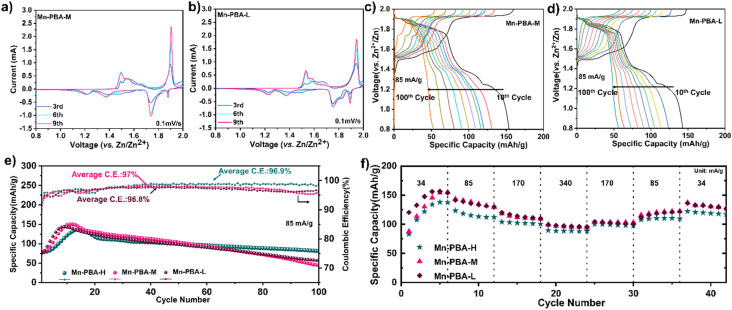
Electrochemical performance of Mn-PBA-M and Mn-PBA-L in 1 M Na_2_SO_4_ + 1 M ZnSO_4_ electrolyte. (a and b) Cyclic voltammetry curves of (a) Mn-PBA-M and (b) Mn-PBA-L recorded at a scan rate of 0.1 mV s^−1^. (c and d) Capacity–voltage profiles over 10–100 cycles illustrating the evolution of redox plateaus for (c) Mn-PBA-M and (d) Mn-PBA-L. (e) Comparison of long-term cycling stability for all Mn-PBA variants. (f) Rate-capability Zn-PBA comparison of the Mn-PBA variants.

Both Mn-PBA-M and Mn-PBA-L exhibit redox features similar to Mn-PBA-H, with cathodic peaks at ∼1.5 V and ∼1.9 V and four anodic peaks at 1.87 V, 1.74 V, 1.34 V, and 1.22 V (Fig. 6a and b). However, Mn-PBA-L shows a weaker current response at ∼1.9 V, indicating reduced electrochemical activity at higher potentials compared to Mn-PBA-M and Mn-PBA-H. Their capacity–voltage profiles (Fig. 6c and d) at 85 mA g^−1^ resemble those of Mn-PBA-H, requiring several activation cycles to reach peak capacities of 149.9 mA h g^−1^(Mn-PBA-M) and 143.7 mA h g^−1^ (Mn-PBA-L), similar to Mn-PBA-H (Fig. 6e). During long-term cycling at 85 mA g^−1^, the specific capacities of Mn-PBA-M and Mn-PBA-L decline to 56.6 mA h g^−1^ and 44.3 mA h g^−1^, respectively. At a lower current of 17 mA g^−1^, activation is quick, with the specific capacity reaching above 150 mA h g^−1^ within the first few cycles for all variants (Fig. S15). However, the capacity fading is much faster at this lower current, with the capacity reaching ∼50 mA h g^−1^ within 50 cycles. Rate capability tests show that both variants maintain stable performance across current densities, with capacities slightly higher than Mn-PBA-H (Fig. 6e).

**Fig. 7:**
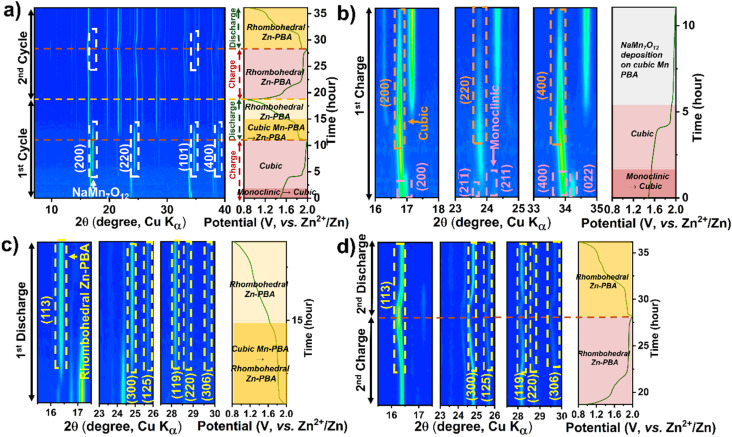
*Operando* XRD contour plots showing the phase evolution of Mn-PBA-L in (a–1 M Na_2_SO_4_ + 1 M ZnSO_4_ electrolyte). (a) Show the first two charge–discharge cycles, (b) the first charge, (c) the first discharge, and (d) the second charge–discharge cycle. Key phase transformations and structural evolutions during cycling are highlighted by arrows, labels, and legends.

*Operando* XRD analysis was further employed to decipher the structural evolution of Mn-PBA-M and Mn-PBA-L in the dual-salt electrolyte. The 2D contour plot for Mn-PBA-L (Fig. 7a) reveals a sequence of transformations of the Mn-PBA framework consistent with Mn-PBA-H. The monoclinic to cubic transition initiates at the onset of charging with a fully developed cubic phase formed by 1.5 V (Fig. 7b). This is followed by deposition of the manganese oxide phase detected at ∼1.8 V. Discharge drives progressive transformation of the cubic Mn-PBA into rhombohedral Zn-PBA, alongside dissolution of the deposited manganese oxide, accelerating below 1.8 V (Fig. 7c). Reversible ion intercalation during the second cycle becomes evident from the distinct shifts in the (113) and (300) diffraction peaks. Mn-PBA-M exhibits an identical structural evolution (Fig. S16), indicating that no qualitative change in the phase transition pathway is observed across the compositions investigated. Neither M nor L Mn-PBA variants change the overall pathway: the same monoclinic → cubic → rhombohedral sequence, Mn dissolution/oxide deposition, and ZHS precipitation are observed. Thus, despite differences in composition, including sodium content, vacancy concentration, and lattice water, all Mn-PBA variants exhibit similar phase evolution pathways, indicating a common underlying reaction mechanism. These observations should be interpreted in the context of the intrinsic coupling between sodium content, vacancy chemistry, and lattice water in PBAs, which prevents strict isolation of individual variables. Within this coupled parameter space, variations in electrochemical response are reflected primarily in activation behavior and reaction kinetics.

### A convergent mechanistic perspective on the electrochemistry

The detection of Mn^2+^ in the electrolyte during charging and early in the discharge confirms manganese dissolution driven by Jahn–Teller distortion, whereas the absence of Fe leaching underscores the stability of the [Fe(CN)_6_]^4−^ framework. This contrast raises a critical question: does Fe actively participate in redox processes in Mn-PBA or remain electrochemically inert during cycling? To address this, soft X-ray absorption spectroscopy (XAS) at the metal L-edge was employed to probe the valence states of Mn and Fe as a function of charge/discharge. The L-edge transitions (2p → 3d) provide fingerprints of charge, spin, and orbital ordering, enabling precise insights into local electronic structure.^41^ XAS measurements were performed in partial electron yield (PEY, ∼2–3 nm), total electron yield (TEY, ∼5–10 nm), and fluorescence yield (FY, ∼50–200 nm) modes to capture surface and bulk characteristics.^42^ Mn-PBA-H electrodes cycled in the dual salt electrolyte at 17 mA g^−1^ were analysed to track oxidation-state evolution.

**Fig. 8:**
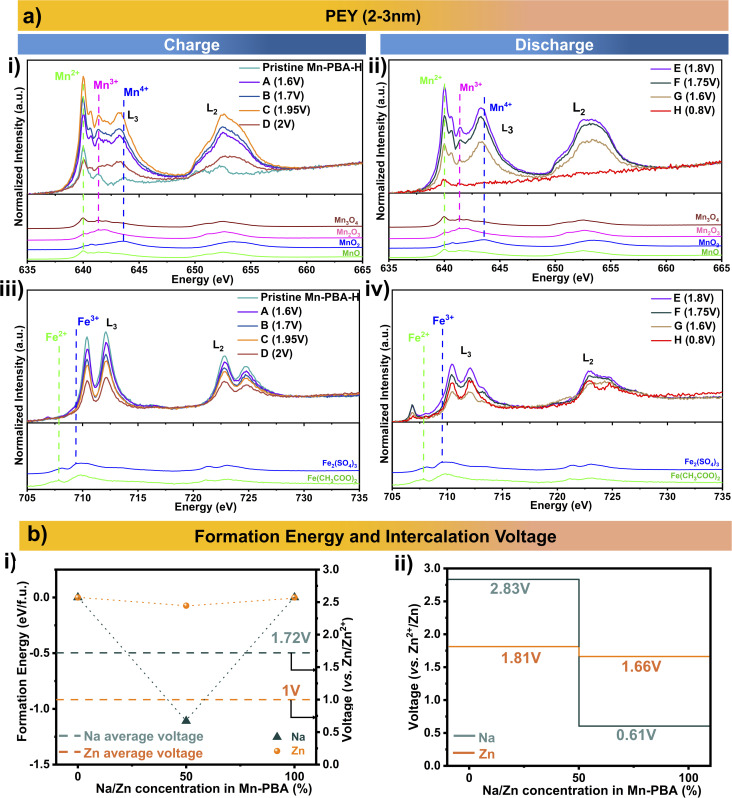
Experimental and computational insights into the redox behaviour of Mn-PBA. (a) Surface sensitive XAS PEY L-edge of Mn and Fe during 1st charge/discharge. (b) Formation energy convex hull for Na^+^ and Zn^2+^ intercalation in Mn-PBA and their corresponding voltage profile.

PEY spectra of the pristine electrode (Fig. 8a(i)) show predominantly Mn^2+^ with minor Mn^3+^/Mn^4+^ contributions, consistent with prior reports.^43^ Upon charging A → C (Fig. S17), the relative intensity of the Mn^2+^ feature decreases, with a concurrent growth of Mn^3+^/Mn^4+^ signals, corroborating deposition of the manganese oxide phase. At the end of the first charge, the surface shows a near equal mix of Mn^2+^, Mn^3+^ and Mn^4+^, consistent with the surface coverage by the NaMn_7_O_12_ phase, potentially accompanied by amorphous or poorly crystalline MnO_*x*_ species, although this remains speculative and is not directly evidenced in the present study. This is further supported by the TEY mode spectra (Fig. S18), which probe slightly deeper into the surface. During discharge (E → G Fig. 8a(ii)), the Mn-L edge signatures corresponding to NaMn_7_O_12_ progressively diminish, consistent with its electrochemical dissolution. At the end of discharge, only the Mn^2+^ signature remains, with a very low Mn L-edge intensity, which can be explained by the dominance of Zn-PBA in the discharged electrode, with some residual untransformed Mn-PBA revealed as surface deposits dissolve. The bulk-sensitive FY spectra (Fig. S19) show a similar evolution of the Mn oxidation states as a function of charge–discharge as in the PEY/TEY mode; however, due to the inherently low fluorescence yield at the Mn L-edge and associated self-absorption effects, the signal intensity from deeper regions is limited, resulting in increased noise in the data. For Fe L-edge (Fig. 8a(iii) and (iv), S18 and S19), Fe^2+^/Fe^3+^ signals appear at a much higher energy than expected (Fe_2_(SO_4_)_3_, Fe(CH_3_COO)_2_), owing to the ligand π* orbital contribution with metal–ligand back-bonding,^42,44^ an effect absent in the reference samples. Nevertheless, the Fe L-edge shows no oxidation-state change during the initial charging in all three modes, confirming non-participation of the iron in the initial redox process, except for a decline in signal intensity in the PEY and TEY modes due to the surface coverage by the manganese oxide phase. Upon discharge, Fe spectra show a distinct evolution with the appearance of a small peak at a lower energy than the Mn-PBA Fe L-edge in both TEY and FY modes. Additionally, the primary L-edge exhibits a distinct evolution in the FY mode. These are likely attributable to the complex multistep transformation of Mn-PBA into Zn-PBA as proposed below.

The electrochemical evolution of Mn-PBAs is intrinsically governed by the strong coupling between structural water, vacancy chemistry, and cation configurations, as evidenced by the correlated variation of vacancy and water content with Na stoichiometry (see Table S1). In particular, lattice water not only elevates intercalation voltages and suppresses volume change, but also alters the phase-transition pathway relative to Na^+^-only intercalation.^45–47^ Notably, Xiao *et al.* reported substantially higher Na-intercalation voltages for hydrated Mn-PBA (3.6 V and 4.15 V *vs.* Na^+^/Na) than for its de-hydrated counterpart (3.36 V and 2.9 V *vs.* Na^+^/Na).^48^ Given this intricate interplay between hydration, vacancies, and cation occupancy, a simplified thermodynamic framework based on de-hydrated cubic Mn-PBA was adopted to evaluate Na^+^/Zn^2+^ intercalation voltages using first-principles calculations and rationalise the proposed mechanism evolution.

Density functional theory (DFT) calculations were conducted to investigate Na^+^ and Zn^2+^ intercalation in cubic Mn-PBA across three representative compositions: the fully de-intercalated state (*x* = 0), half-intercalated state (*x* = 50%), and fully-intercalated state (*x* = 100%) (Fig. S20). For Na insertion, these correspond to MnFe(CN)_6_, Na_1_MnFe(CN)_6_, and Na_2_MnFe(CN)_6_, whereas Zn insertion yields MnFe(CN)_6_, Zn_0.5_MnFe(CN)_6_, and Zn_1_MnFe(CN)_6_. For the intermediate composition (*x* = 50%), the configuration with the lowest Ewald energy was selected to represent the thermodynamically most stable structure.

The calculated structural evolution reveals a pronounced lattice contraction upon progressive Na insertion (volume contraction of 10.32% and 13.83% for *x* = 50% and *x* = 100%, respectively), whereas the contraction associated with Zn insertion is comparatively smaller (volume contraction of 5.2% and 4.46% for *x* = 50% and *x* = 100%, respectively) (Table S2). At a low Na concentration, Pauli repulsion between Na^+^ ions and the host framework induces a slight lattice expansion. However, once the framework is partially filled, coulombic attractions between the intercalated cations and the negatively charged framework dominate, leading to an overall contraction.^49^ In contrast, the higher charge density of Zn^2+^ generates stronger electrostatic interactions within the framework, thereby moderating the extent of lattice shrinkage.^50^

Formation energy analysis indicates a thermodynamically stable intermediate phase at *x* = 50% for both Na and Zn insertion (Fig. 8b(i)), giving rise to a convex hull and a two-step intercalation pathway. Accordingly, Na intercalation proceeds through sequential transitions MnFe(CN)_6_ → Na_1_MnFe(CN)_6_ → Na_2_MnFe(CN)_6_, with deintercalation following the reverse pathway. A similar mechanism is observed for Zn insertion, proceeding through MnFe(CN)_6_ → Zn_0.5_MnFe(CN)_6_ → ZnMnFe(CN)_6_.

The calculated average intercalation voltage for Na is 1.72 V *vs.* Zn/Zn^2+^, whereas Zn insertion exhibits a lower average voltage of 1 V *vs.* Zn/Zn^2+^, indicating that Na insertion is thermodynamically more favourable within the Mn-PBA framework. Consistent with the presence of a stable intermediate phase, both systems exhibit two voltage plateaus (Fig. 8b(ii)). For Na insertion, the higher plateau at 2.83 V (*vs.* Zn/Zn^2+^) corresponds to the first Na insertion per formula unit, while the lower plateau at 0.63 V (*vs.* Zn/Zn^2+^) corresponds to the second Na insertion, resulting in full sodiation. In contrast, Zn insertion exhibits plateaus at 1.81 V (*vs.* Zn/Zn^2+^) and 1.66 V (*vs.* Zn/Zn^2+^), corresponding to the first and second half-equivalent Zn insertion, respectively. Notably, the voltage required to insert Na from the fully de-intercalated state to the half-filled state is higher than that of Zn, suggesting a stronger thermodynamic driving force for Na insertion during the initial stage of intercalation. However, beyond the half-filled state, the intercalation voltage of Zn exceeds that of Na, indicating that further Zn insertion becomes comparatively more favourable at higher occupancy of the framework. This theoretical insight aligns well with the experimental observations. In the single-salt electrolyte, the discharge process is dominated by Zn^2+^ insertion, where the influx of Zn^2+^ into the Mn-PBA framework promotes structural reconstruction and a progressive transformation into Zn-PBA, ultimately accelerating cathode degradation. In contrast, in the dual salt electrolyte, the presence of Na^+^ alters the intercalation thermodynamics. The relatively favourable driving force for Na^+^ insertion at higher discharge potentials enables partial Na^+^ intercalation, which competes with Zn^2+^ insertion and mitigates rapid framework reconstruction. As a result, the transformation of Mn-PBA into Zn-PBA is significantly delayed, stabilising the cathode structure and slowing the degradation process.

To further probe the redox chemistry of the framework, the evolution of the Mn oxidation state during Na/Zn intercalation was analysed (Fig. S21). The calculated average oxidation state of Mn remains within the +2 to +3 range throughout the (de)intercalation process, consistent with the Mn^2+^/Mn^3+^ redox couple. This oxidation state window is also indicative of potential Mn disproportionation reactions within the framework, which contribute to the Mn dissolution observed experimentally.

### Integrated mechanistic picture

**Fig. 9:**
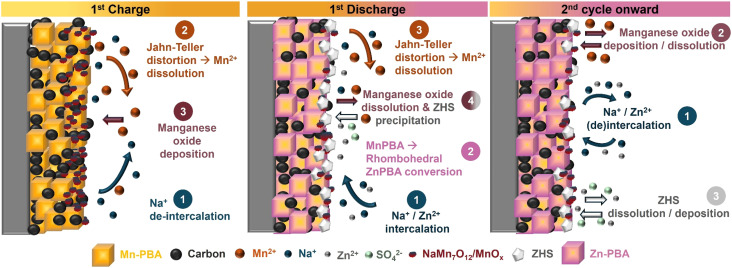
Schematic of the overall charge-storage mechanism of Mn-PBA in aqueous zinc-ion electrolytes. The morphologies shown here are illustrative and do not represent the actual shapes or sizes of particles from different phases. H^+^, involved in Mn oxide dissolution and the associated OH^−^, consumed during ZHS formation, are not depicted. The initial monoclinic-to-cubic transition of Mn-PBA upon Na^+^ extraction is also omitted. The gradient shading in the PBA cubes indicates that some pristine Mn PBA (light yellow) may persist in the particle core and can require several cycles to fully convert, depending on the applied current.

The overall processes occurring during charge and discharge can be summarized as follows (Fig. 9). On the first charge, Na^+^ deintercalation drives a monoclinic to cubic transition (∼1.6 V), creating Mn^3+^-rich, Jahn–Teller destabilized surface regions that undergo disproportionation with Mn^2+^ release; above ∼1.8 V, dissolved Mn^2+^ electrochemically deposits as NaMn_7_O_12_/MnO_*x*_, providing capacity beyond the 1-electron limit of the host (steps C1–C3). During the first discharge, the cubic Mn-PBA converts to rhombohedral Zn–PBA, potentially *via* a transient, partially soluble intermediate, although this remains inferred and is not directly evidenced in the present study – while the manganese oxide dissolves *via* reduction (D1–D3); concomitant H^+^ consumption raises local pH near the cathode and precipitates ZHS, which later dissolves upon acidification or H^+^ release during the next charge. In the dual-salt electrolyte, higher Na^+^ activity/ionic strength (i) delays Mn dissolution, (ii) allows OCV-driven cubic → rhombohedral drift, and (iii) stabilizes partial (de)intercalation within Zn–PBA; in the single-salt electrolyte, the cubic → rhombohedral conversion is strictly potential-driven and intercalation signatures for Zn–PBA are weak. Across Na-stoichiometries (H/M/L), the qualitative pathway is invariant, indicating that electrolyte-controlled interfacial chemistry – not initial lattice Na – can govern durability.

1st charge:C1: Na_2_MnFe(CN)_6_ (s) → NaMnFe(CN)_6_ (s) + Na^+^ (aq) + e^−^ (Na^+^ de-intercalation process)

The Mn^3+^ in the lattice undergoes Jahn–Teller distortion-mediated destabilization, and Mn^2+^ dissolution takes place.
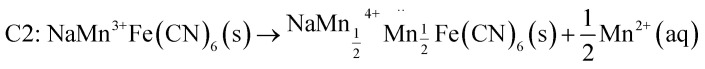
Here, 
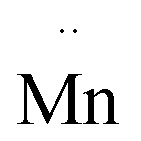
 is the Mn vacancy in the lattice.C3: Na^+^ (aq) + 7Mn^2+^ (aq) + 12H_2_O (l) → NaMn_7_O_12_ (s) + 24H^+^+ 9e^−^

1st discharge: 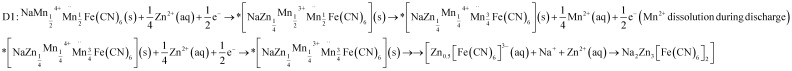
D2: NaMn_7_O_12_ (s)+ 24H^+^ + 9e^−^ → Na^+^ (aq) + 7Mn^2+^ (aq) + 12H_2_O (l)D3: 4Zn^2+^ (aq) + SO_4_^2−^ (aq) + 5H_2_O (l) + 6OH^−^ → Zn_4_SO_4_(OH)_6_·5H_2_O (s)

## Conclusion

Comparing single-salt and dual-salt electrolytes while tuning lattice Na establishes that Mn-PBAs in AZIBs operate *via* a dissolution–redeposition-assisted conversion pathway: (i) monoclinic → cubic transition and surface Mn^3+^ disproportionation on the first charge, (ii) NaMn_7_O_12_ (with a possible contribution from amorphous MnO_*x*_) deposition providing capacity beyond 1-electron, (iii) cubic → rhombohedral Mn-PBA to Zn–PBA conversion on the first discharge with ZHS formation, and (iv) subsequent cycling dominated by Zn–PBA solid-solution behavior along with repeated oxide deposition – dissolution. The Na^+^–Zn^2+^ dual-salt electrolyte delays Mn dissolution and enables partial (de)intercalation within Zn–PBA as well as OCV-driven cubic-rhombohedral phase drift, whereas the Zn^2+^ based single-salt electrolyte cells require an applied potential for conversion and show minimal intercalation signatures for the Zn-PBA. Fe remains largely redox-inactive during the initial charge, with only minor spectral evolution observed during discharge, likely associated with the Mn-PBA to Zn-PBA transformation and accompanying structural reorganization.

More broadly, these findings resolve persistent ambiguity in the Mn-PBA literature regarding the origin of capacity and apparent stability in AZIBs. Previous reports attributing enhanced cycling performance to alkali stoichiometry, lattice water content, or electrolyte formulation often rely on electrochemical metrics alone, without resolving phase evolution or the identity of the active material during cycling. Our results demonstrate that such improvements predominantly reflect kinetic suppression or delayed manifestation of Mn dissolution and framework conversion, rather than stabilization of reversible Mn-PBA redox. In this context, reports of prolonged cycling stability at high rates, often accompanied by relatively low specific capacities, are most consistently explained by the emergence and subsequent cycling of Zn-PBA and manganese oxide type phases, rather than sustained electrochemical activity of the parent Mn-PBA framework.

Taken together, this study establishes a fundamental limitation of Mn-PBAs in aqueous Zn electrolytes: compositional tuning of the host lattice or modest electrolyte modifications cannot, on their own, overcome Mn^3+^ disproportionation and proton-coupled dissolution. Future strategies must therefore move beyond stoichiometric optimization and instead focus on directly stabilizing the Mn redox chemistry at the electrode–electrolyte interface, for example through interphase engineering, suppression of Mn dissolution, regulation of local pH and anion chemistry, or electrolyte designs that decouple charge compensation from framework breakdown. Addressing these challenges is essential for translating the structural versatility of PBAs into genuinely durable cathodes for aqueous Zn-ion batteries.

## Experimental section

### Synthesis of different variants of sodium Mn-PBA and sodium Zn-PBA

Sodium Mn-PBAs were synthesised through a simple, reproducible co-precipitation method. Manganese(ii) acetate tetrahydrate [Mn(CH_3_COO)_2_·4H_2_O] (Sigma Aldrich), sodium-EDTA(C_10_H_14_N_2_Na_2_O_8_.2H_2_O) (Sigma Aldrich) and sodium ferrocyanide decahydrate [Na_4_Fe(CN)_6_.10H_2_O] (Sigma Aldrich) were used as precursors in a molar ratio of 1 : 1 : 1. To modulate sodium content in the final product, varying concentrations of sodium chloride (NaCl) were introduced during synthesis. Two separate precursor solutions were prepared: solution A, containing 0.04 M Mn(CH_3_COO)_2_·4H_2_O and 0.04 M Na-EDTA and solution B, containing 0.04 M Na_4_Fe(CN)_6_·10H_2_O along with NaCl at different concentrations for the three targeted compositions – 3 M for Mn-PBA-H (high sodium), 1.5 M for Mn-PBA-M (medium sodium) and 1 M for Mn-PBA-L (low sodium). Solution A was added to solution B at a controlled rate of 5 ml min^−1^ using a peristaltic pump under a nitrogen atmosphere and continuous stirring at room temperature. Following the addition, the precipitate was aged for 24 hours in a nitrogen environment without agitation, allowing for complete crystallisation. The resulting precipitate was filtered, washed thoroughly with deionised water and ethanol to remove residual precursors, and subsequently dried under vacuum at 80 °C overnight. Sodium Zn-PBA was synthesised following the same procedure, with the only modification being the replacement of manganese acetate with 0.04 M zinc acetate dihydrate [Zn(CH_3_COO)_2_·2H_2_O] (Sigma Aldrich) in solution A.

### Physicochemical and morphostructural characterisations

Elemental analysis of the PBAs, cycled electrode, and electrolytes was carried out using Inductively Coupled Plasma Optical Emission Spectroscopy (ICP-OES, AVIO-560 DV, PerkinElmer). Before the measurement, samples were digested in a Multiwave GO microwave digestion system (Anton Paar) to ensure complete dissolution of the constituents. The elemental composition (C, H, N, and S) of the as-prepared PBAs was determined using a Vario MACROCUBE elemental analyser to assess the framework integrity and compositional uniformity.

X-ray diffraction (XRD) was performed on both pristine powders and electrode samples using PANanalytical Empyrean-I and Empyrean IV diffractometers operated in Bragg–Brentano geometry using Cu Kα_1_ radiation (*λ* = 1.540598 Å), a PIXcel detector and a Ni Kβ_1_ filter. Diffraction patterns were collected over a 2*θ* range of 5°–60° at a scan rate of 0.007° per second. *Operando* XRD experiments were conducted on a PANanalytical Empyrean-II diffractometer under identical optical configurations, with a time resolution of 15 minutes per scan to monitor structural evolution during cycling.

Morphological and compositional characterisation was conducted using field-emission electron microscopy (FE-SEM, Nova NanoSEM 450, FEI) equipped with an energy-dispersive X-ray spectroscopy (EDS) detector. For SEM imaging, powder samples were dispersed in ethanol, drop-cast onto silicon substrates, and dried under ambient conditions. Cycled electrodes were rinsed thoroughly with deionised water and ethanol, then dried. To remove surface-deposited ZHS from discharged electrodes, a mild wash with 0.01 M H_2_SO_4_ was performed before standard cleaning. All samples were affixed onto carbon tape and coated with a ∼10 nm layer of platinum using a sputter coater to minimise charging under the electron beam.

High-resolution transmission electron microscopy (HR-TEM), selected area electron diffraction (SAED), and energy-dispersive X-ray spectroscopy (EDS) were conducted using JOEL JEM-F200 S/TEM operated at 200 kV. A dilute suspension of the as-prepared powder (Mn-PBA-H) and that of the cycled electrodes were prepared in ethanol and sonicated to achieve uniform dispersion. An aliquot of the suspension was drop-cast onto the shiny side of a 200-mesh holey carbon grid to ensure good adhesion to the carbon film. The grids were dried overnight at room temperature in a dust-free environment to allow complete solvent evaporation. Imaging was performed in both TEM and STEM modes to examine the morphology, crystallinity and elemental distribution. EDS spectra were acquired in both secondary and backscattered electron modes at accelerating voltages of 10–15 kV. The microscope is equipped with a 100 mm^2^ silicon drift detector to enable high-sensitivity elemental mapping.

Fourier-Transform Infrared (FTIR) spectra of the Na–Mn PBAs were collected using a Spectrum100 spectrometer in the range of 40 000–1000 cm^−1^.

Thermogravimetric analysis (TGA) was performed on a Q5000 (TA Instruments) under nitrogen flow, with a temperature ramp from room temperature to 400 °C at a heating rate of 5 °C min^−1^.

### Electrode preparation, cell assembly and electrochemical measurements

The working electrode was fabricated by mixing active material, Super P Carbon (TIMCAL), and a binder system comprising Carboxymethyl Cellulose (CMC) and Styrene-Butadiene Rubber (SBR) in a 3 : 2 weight ratio. The overall composition of the electrode slurry was maintained at 70 : 25 : 5 [active material : carbon : CMC-SBR (2.5 wt% of 3 : 2 CMC : SBR)]. A minimal amount of deionised water was added to form a homogeneous and viscous slurry. The resulting slurry was uniformly cast onto a graphite foil (30 µm thickness) and dried in a hot air oven at 60 °C for 12 hours. Circular electrodes with a diameter of 1 cm^2^ were punched from the dried film and used as positive electrode (cathode). The average mass loading of active material was approximately 2–3 mg per electrode. For the negative electrode, zinc metal foil (50 µm thickness) was punched into 1 cm^2^ disks and used directly. *Operando* XRD measurements were conducted using a glassy carbon disk (0.78 cm^2^ area, 60 µm thickness) as the current collector, which was coated with the same slurry. A zinc foil disk (0.64 cm^2^, 20 µm thickness) served as the negative electrode in this configuration. The average active material loading on the glassy carbon window was approximately 8–9 mg. Glass fibre separator pre-soaked in electrolyte (50 µL for Swagelok cells, 90 µL for *operando* cells) was placed between the electrodes. Swagelok cells used a single Merck Millipore separator (thickness 380 µm, pore size 0.7 µm) while *operando* cells employed a stack of two Merck Millipore and one Whatman GF/D separator (thickness ∼670 µm, pore size 2.7 µm). Two variants of the electrolytes *viz* 1 M Na_2_SO_4_ + 1 M ZnSO_4_ and 1 M ZnSO_4_ were prepared by adding the calculated amount of salts in Milli-Q water.

Galvanostatic cycling was carried out in two-electrode Swagelok-type cells using titanium current collectors and operated on a VMP3 potentiostat/galvanostat (BioLogic) or a LAND CT2001A battery tester within a potential window of 0.8–2 V *vs.* Zn. For *operando* investigation, a custom-designed in-house titanium body cell equipped with a glassy carbon window was employed, and the cycling was performed using a portable PalmSens4 potentiostat/galvanostat. All electrochemical measurements were conducted at room temperature.

### DFT methodology

In this study, spin-polarised first-principles calculations were employed to investigate the intercalation behaviour of A_*x*_MnFe(CN)_6_ (where A = Na or Zn) using the Vienna *Ab Initio* Simulation Package (VASP)^51,52^ within the framework of Density Functional Theory (DFT). The Projector Augmented Wave (PAW)^53^ method and a plane-wave basis set were used for the calculations. The Generalised Gradient Approximation (GGA), in the form of the Perdew–Burke–Ernzerhof (PBE)^54^ functional, was used in conjunction with the Hubbard U correction (GGA + U), following the approach of Dudarev, to better describe the localised 3d electrons in transition metals. Although the Heyd–Scuseria–Ernzerhof (HSE06) hybrid functional provides an accurate description of PBA systems, previous studies have shown that the GGA + U method, with properly calibrated U values, yields results that are quantitatively consistent with those of HSE06.^55^ Consequently, all calculations primarily employed the GGA + U scheme, using U values recommended by the Material Project^56^ database for transition-metal oxides (Mn = 3.9; Fe = 5.3). Given that the d^10^ orbital of Zn is filled and exhibits negligible on-site electron correlation, no U correction was applied to it.

The intercalation behaviour of A_*x*_MnFe(CN)_6_ was examined in the context of cubic crystal symmetry. The corresponding primitive unit cells were obtained from the Inorganic Crystal Structure Database (ICSD). Geometrical optimisation calculations were carried out using the plane-wave cut-off energy of 520 eV, while the electronic and ionic relaxation criteria were set to 10^−5^ eV and 0.05 eV Å^−1^, respectively. 2 × 2 × 2 Monkhorst–Pack *K*-mesh sampling was considered to ensure accurate Brillouin zone mapping. For the composition (*x* = 50% and *x* = 100%), the configuration with the lowest Ewald energy was selected to represent the thermodynamically most stable structure. The formation energies of the compound at different Na/Zn concentrations were calculated using:

For Na^+^:
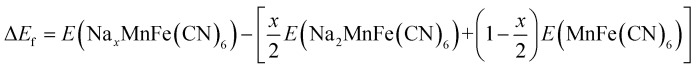


For Zn^2+^:
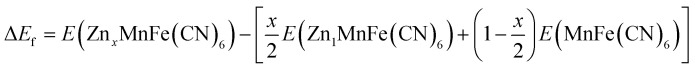
where *E* denotes the total cohesive energy, *E*(MnFe(CN)_6_) serve as the energy reference for the fully de-intercalated structure, *E*(Na_2_MnFe(CN)_6_) and *E*(Zn_1_MnFe(CN)_6_) serve as the energy references for the fully intercalated structures for Na and Zn, respectively.

The average intercalation voltage profile with respect to metallic anodes, specifically (Na/Na^+^) and (Zn/Zn^2+^), is quantitatively calculated by the following relation and then transposed to that with respect to the Zn/Zn^2+^ voltage:
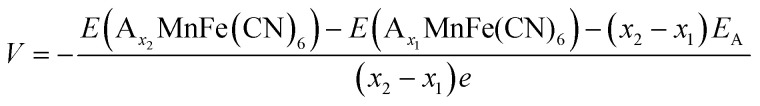
where *V* represents the average voltage, *x*_1_ and *x*_2_ (*x*_2_ > *x*_1_) are the number of Na/Zn ions intercalated/de-intercalated, while *E* and *E*_A_ denote the total energy and energy per atom in the corresponding metallic phase, respectively.

For oxidation state calculation, Bader Analysis^57^ was used. For MnO_2_, Mn_2_O_3_ and Mn_3_O_4,_ relevant cubic phase structures were obtained from the Materials Project.^56^

## Author contributions

S. S.: methodology, investigation, data curation, formal analysis, validation, writing – original draft; Y. S.: methodology, supervision, review/editing of the original draft; R. M.: theoretical investigation; X. Z.: TEM investigation and analysis; P. K.: theoretical investigation methodology, supervision, review/editing of theoretical section writing, funding acquisition. D. K.: conceptualization, methodology, resources, writing – original draft and review & editing, funding acquisition.

## Conflicts of interest

The authors declare no conflicts of interest.

## Supplementary Material

SC-017-D6SC02408D-s001

## Data Availability

Key data presented in this study are available at: https://github.com/sankhadip-saha/Mn-PBA-data.git. Supplementary information (SI): additional experimental and computational details, including compositional analysis, crystallographic refinement, morphology and elemental mapping, electrochemical performance across electrolytes, operando XRD and XAS studies of structural and redox evolution, post-cycling dissolution/morphology analysis, benchmarking against Zn-PBA, and DFT-based insights into lattice changes and oxidation states. See DOI: https://doi.org/10.1039/d6sc02408d.
