# Supplementary material for: Unmasking the intrinsic mechanistic limits of manganese Prussian blue analogues in aqueous Zn-ion batteries
Source: Chem Sci. 2026 May 20;17(26):13155–70. doi: 10.1039/d6sc02408d (PMC13216819; doi:10.1039/d6sc02408d)
Supplement: SC-017-D6SC02408D-s001 [file SC-017-D6SC02408D-s001.pdf]

This journal is © The Royal Society of Chemistry 2026

Unmasking the Intrinsic Mechanistic Limits of Manganese Prussian Blue Analogues in Aqueous Zn-Ion Batteries

Sankhadip Saha,[a] Yuan Shang,[a,] Ruichen Ma,[b] Xiaoran Zheng,[c], Priyank Kumar,[b,*] Dipan Kundu[a,*]*

[a]*LBRI, School of Chemical Engineering, UNSW Sydney, Kensington, NSW 2052, Australia*
[b]*School of Chemical Engineering, UNSW Sydney, Kensington, NSW 2052, Australia*
[c]*School of Chemistry, UNSW Sydney, Kensington, NSW 2052, Australia*

Corresponding authors
Yuan Shang: yuan.shang@unsw.edu.au
Priyank Kumar: priyank.kumar@unsw.edu.au
Dipan Kundu; Email: d.kundu@unsw.edu.au

Table S1 Composition of the three sodium variants calculated using ICP-OES and TGA

	Na	Mn	Fe	C	N	xH_2O
Mn-PBA-H	1.96	1	1	6	6	2.37
Mn-PBA-M	1.79	1	0.93	5.58	5.58	2.67
Mn-PBA-L	1.35	1	0.83	4.96	4.98	3.06

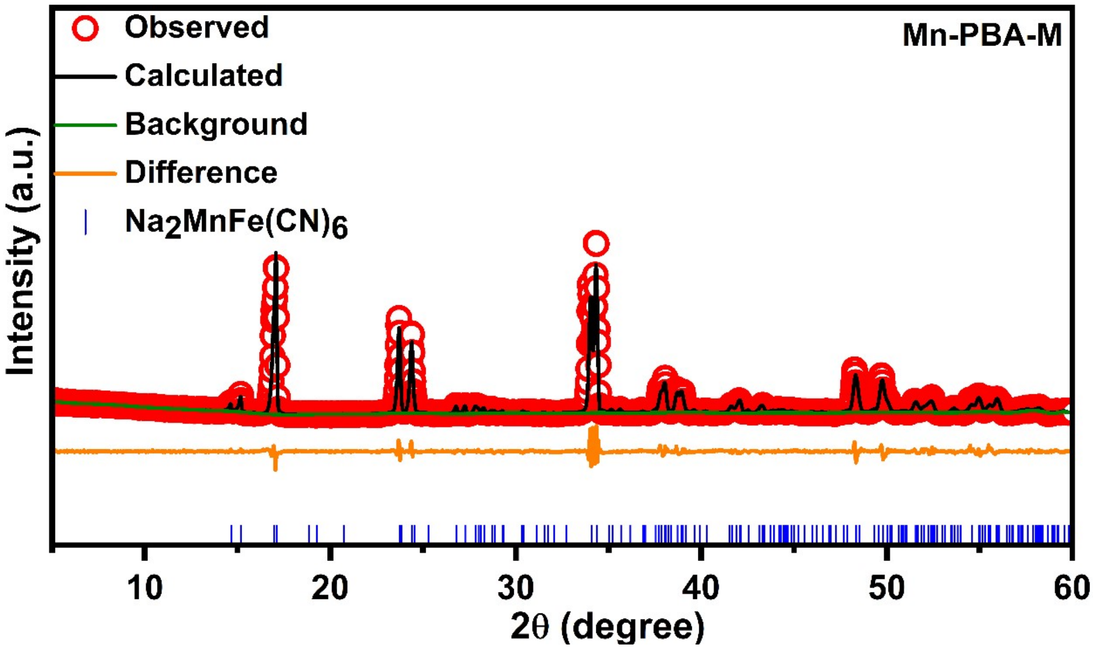

Figure S1. Rietveld refinement (R_{wp}:3.26%, χ^2(gof): 2.97) of the laboratory powder XRD data of as-prepared Mn-PBA-M, using ICSD reference #266392, confirms monoclinic (P*21/n*) phase. Observed data points (o symbol); calculated profile (black line); difference profile (orange line); Bragg positions (blue ticks)

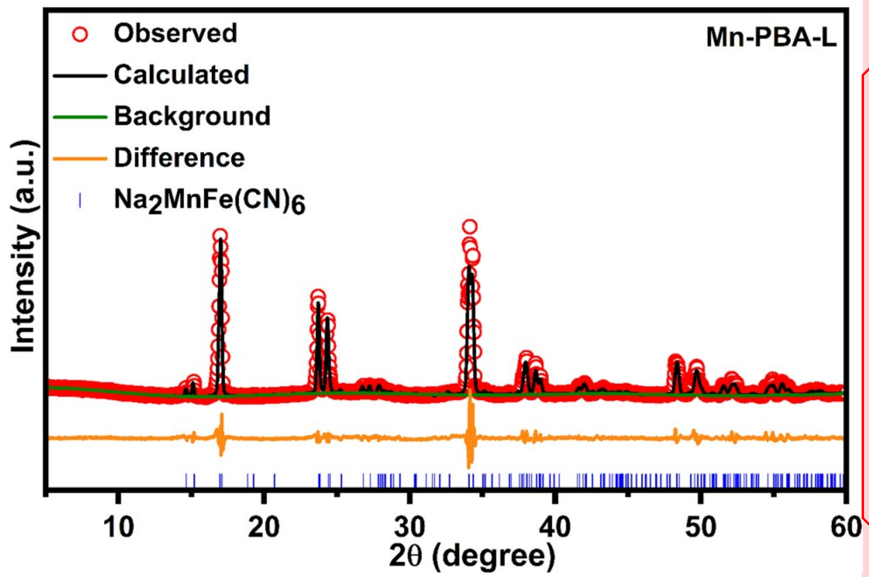

Figure S2. Rietveld refinement (R_{wp}:3.87%, χ^2(gof):3.82) of the laboratory powder XRD data of as-prepared Mn-PBA-L, using ICSD reference #266392, confirms monoclinic (P*21/n*) phase. Observed data points (o symbol); calculated profile (black line); difference profile (orange line); Bragg positions (blue ticks)

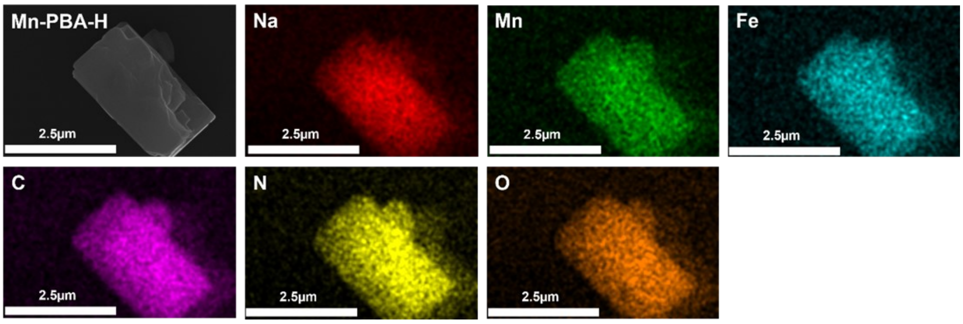

Figure S3. FESEM image illustrating a representative Mn-PBA-H particle accompanied by EDS elemental mapping confirming the uniform distribution of the constituent elements throughout the particles.

Comment [DK]: Are we going to add this plot?

Comment [SS]: Added

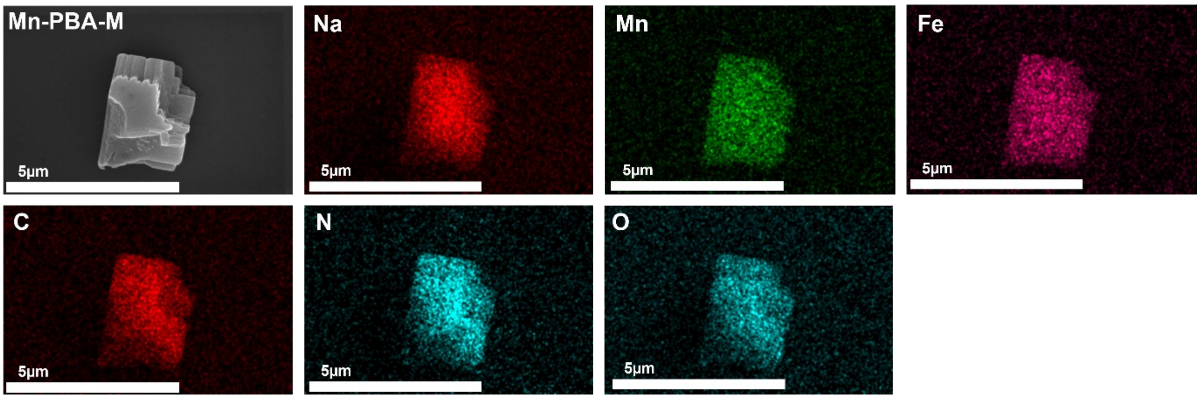

Figure S4. FESEM image illustrating a representative Mn-PBA-M particle accompanied by EDS elemental mapping confirming the uniform distribution of the constituent elements throughout the particles.

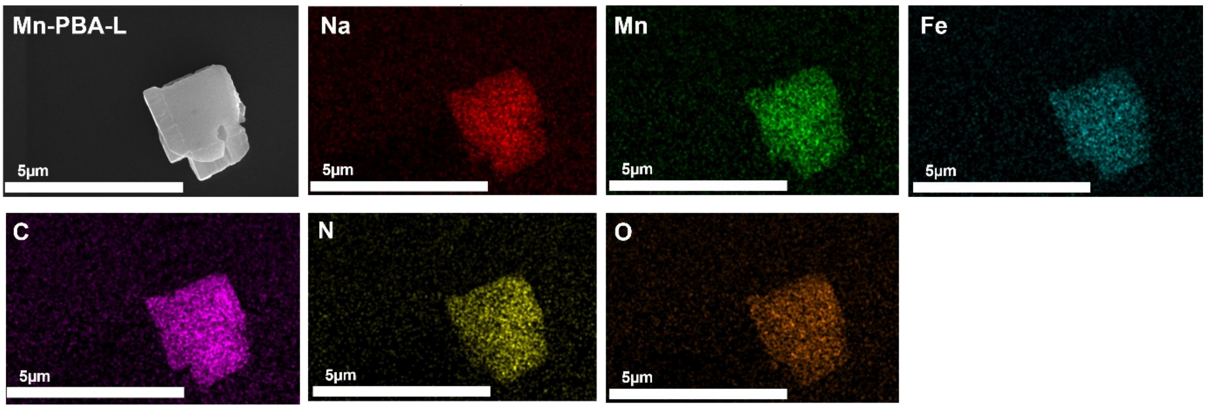

Figure S5. FESEM image illustrating a representative Mn-PBA-L particle accompanied by EDS elemental mapping confirming the uniform distribution of the constituent elements throughout the particles.

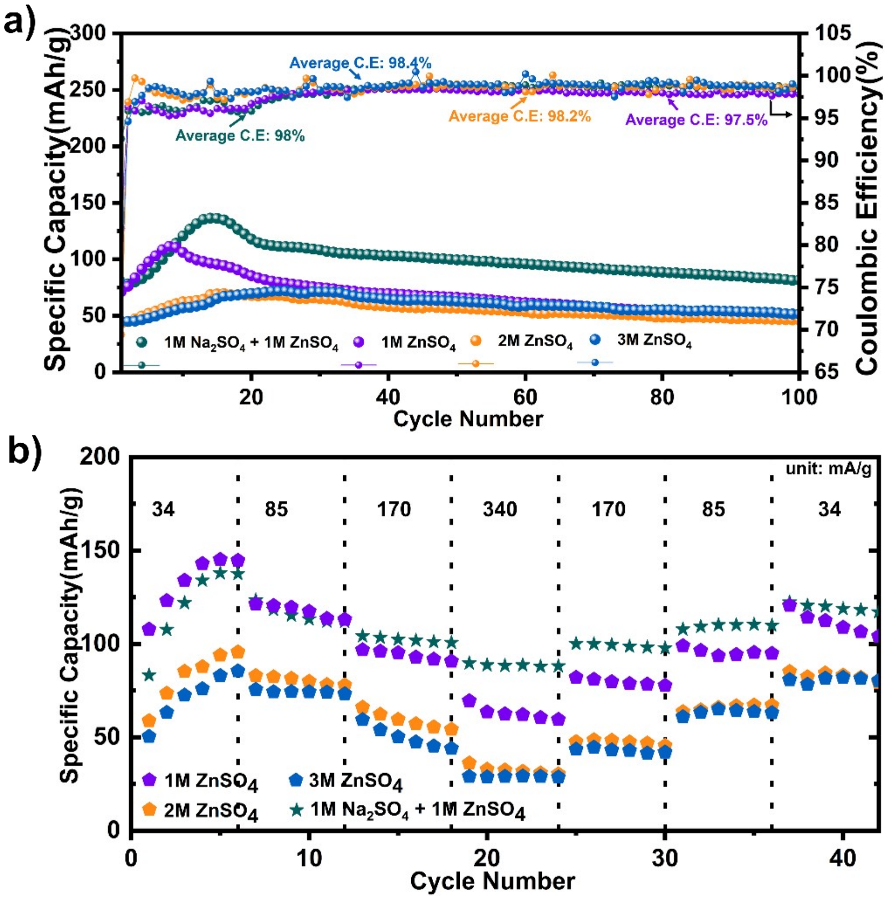

Figure S6 (a) Long-term cycling stability of Mn-PBA-H in 1M Na_2SO_4+1M$ZnSO_4$ and different concentrations of 1M $ZnSO_4$ (1M,2M and 3M). (g) Rate-capability comparison of Mn-PBA-H between the electrolytes.

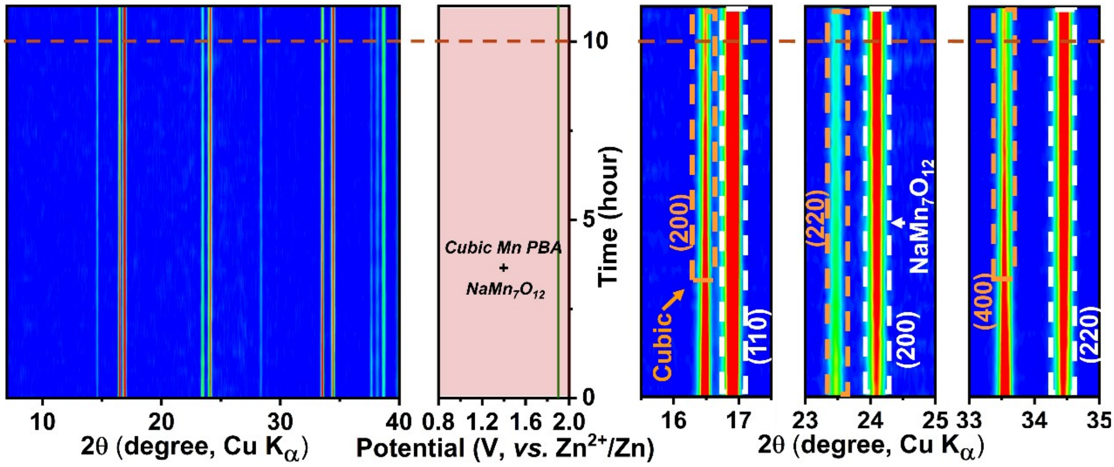

Figure S7 Operando XRD showing the structural evolution of Mn-PBA-H in 1M $ZnSO_4$ holding at OCV after 1st charge.

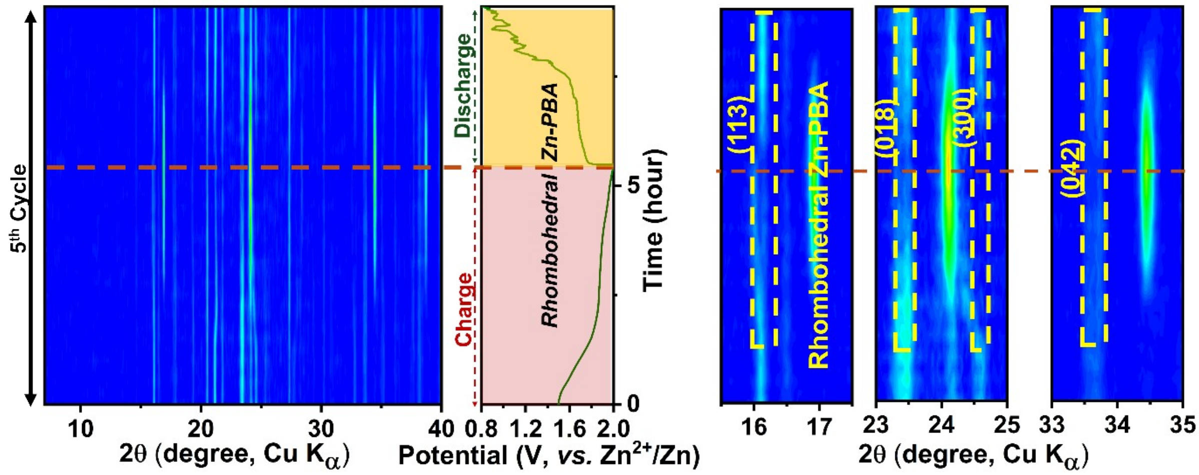

Figure S8 Operando XRD showing the structural evolution of Mn-PBA-H in 1M $ZnSO_4$ during the 5th charge-discharge cycle.

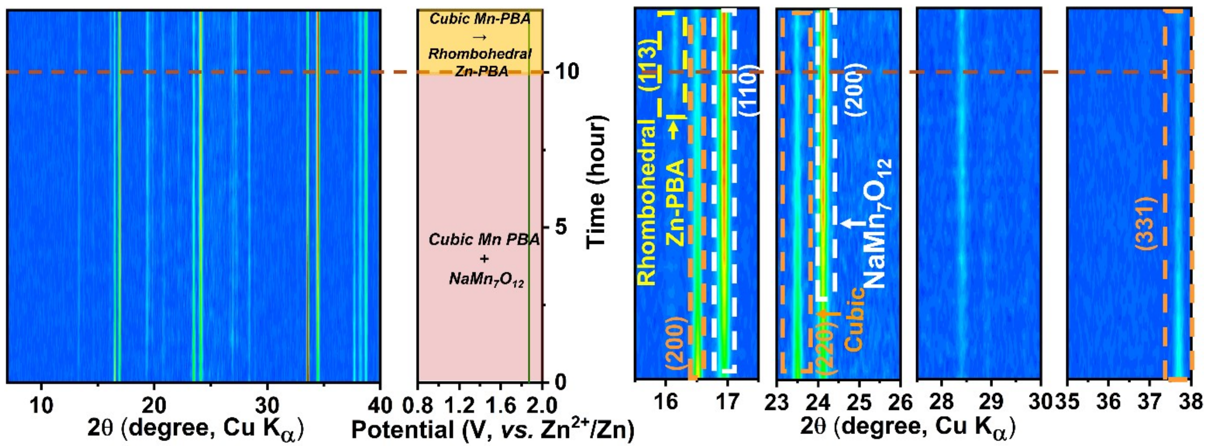

Figure S9 Operando XRD showing the structural evolution of Mn-PBA-H in 1M $ZnSO_4$ + 1M Na_2SO_4 holding at OCV after 1st charge.

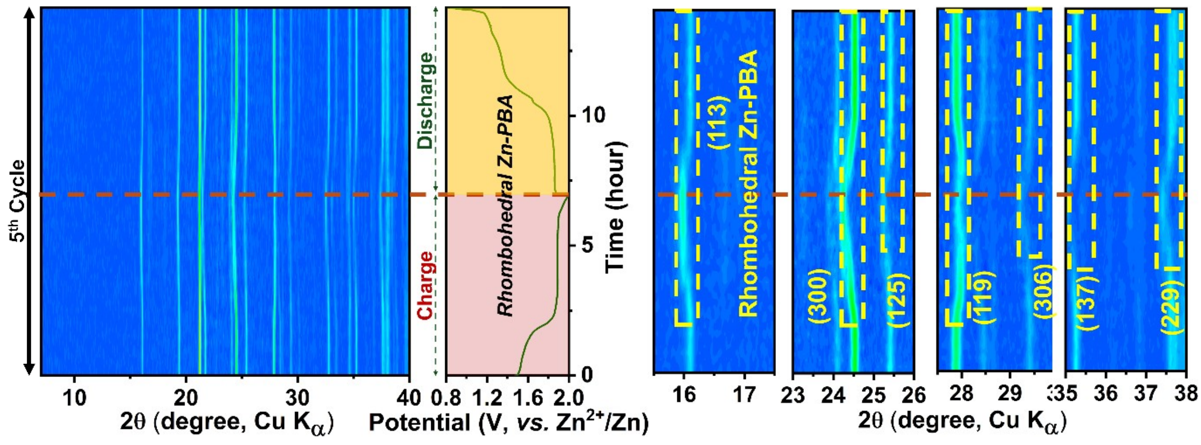

Figure S10 Operando XRD showing the structural evolution of Mn-PBA-H in 1M $ZnSO_4$ + 1M Na_2SO_4 during the 5th charge-discharge cycle.

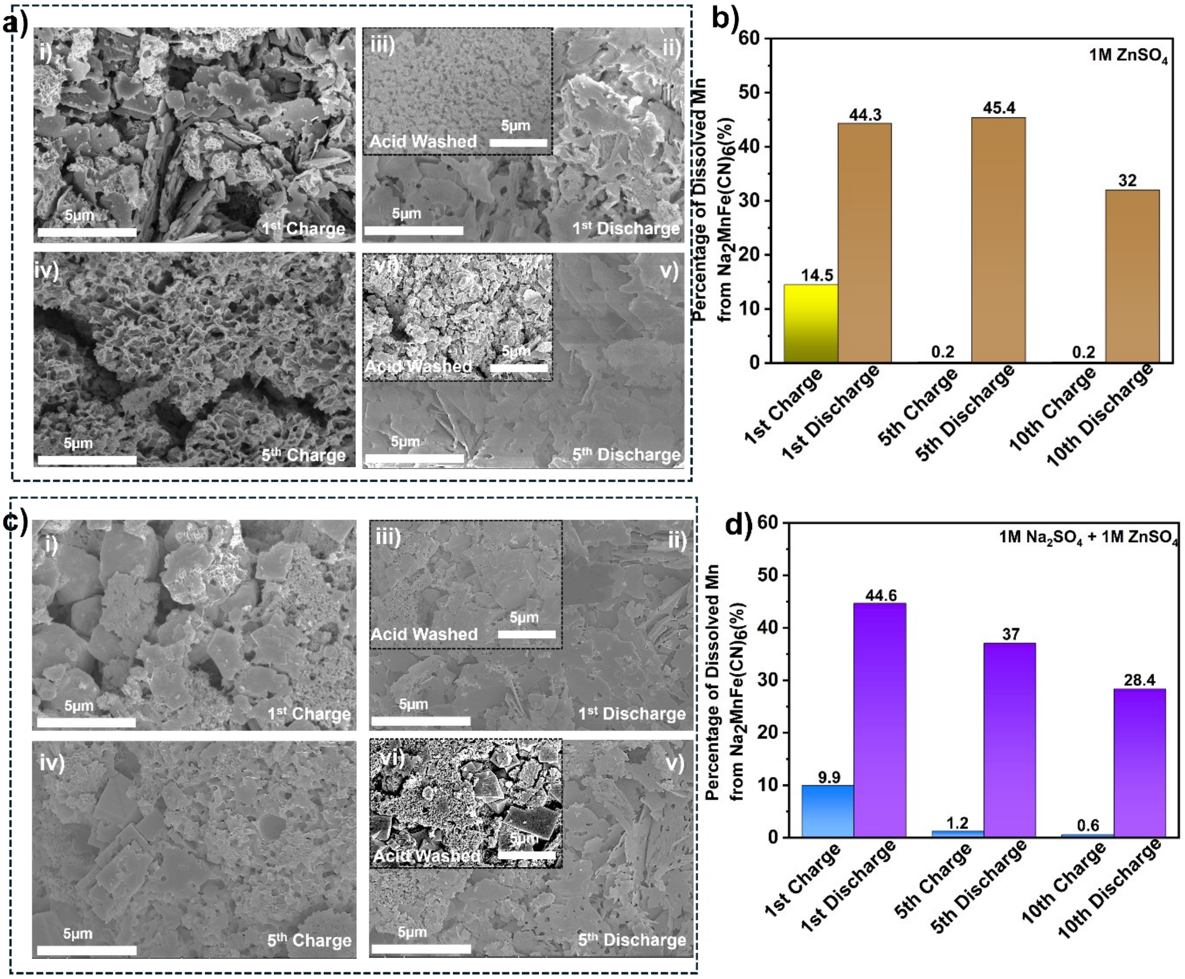

Figure S11. Electrode morphology after cycling in (a-b)1M $ZnSO_4$ and (c-d)1M$ZnSO_4$+1MNa_2SO_4 after 1st and 5th charge-discharge cycle alongside their respective Mn^{2+} concentration analysed after 1st, 5th and 10th charge-discharge cycle, confirming the dissolution-redeposition process.

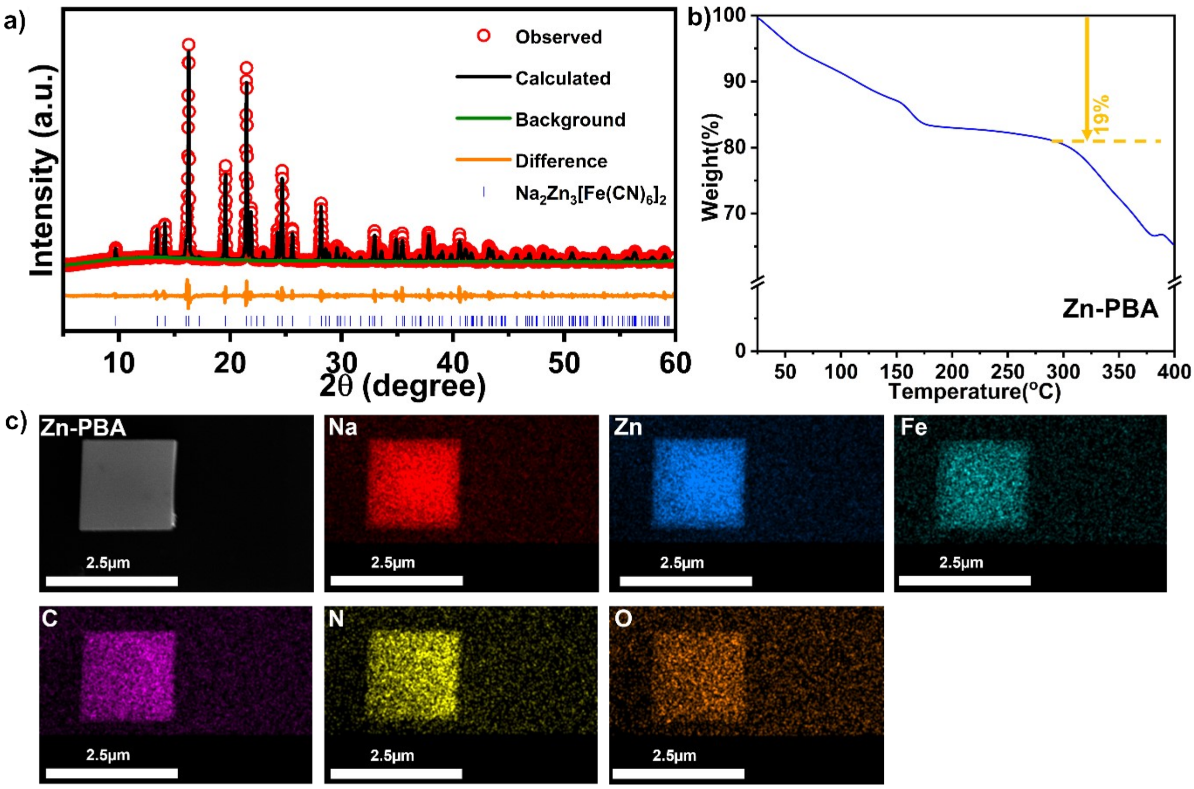

Figure S12. Rietveld refinement (R_{wp}:3.0%. χ^2(gof):3.91) of the laboratory powder XRD data of as-prepared Zn-PBA, using ICSD reference #266392, confirms rhombohedral (R3c: H) phase. Observed data points (o symbol); calculated profile (black line); difference profile (orange line); Bragg positions (blue ticks); TGA showing the interstitial water while EDS confirms uniform elemental distribution throughout the particle.

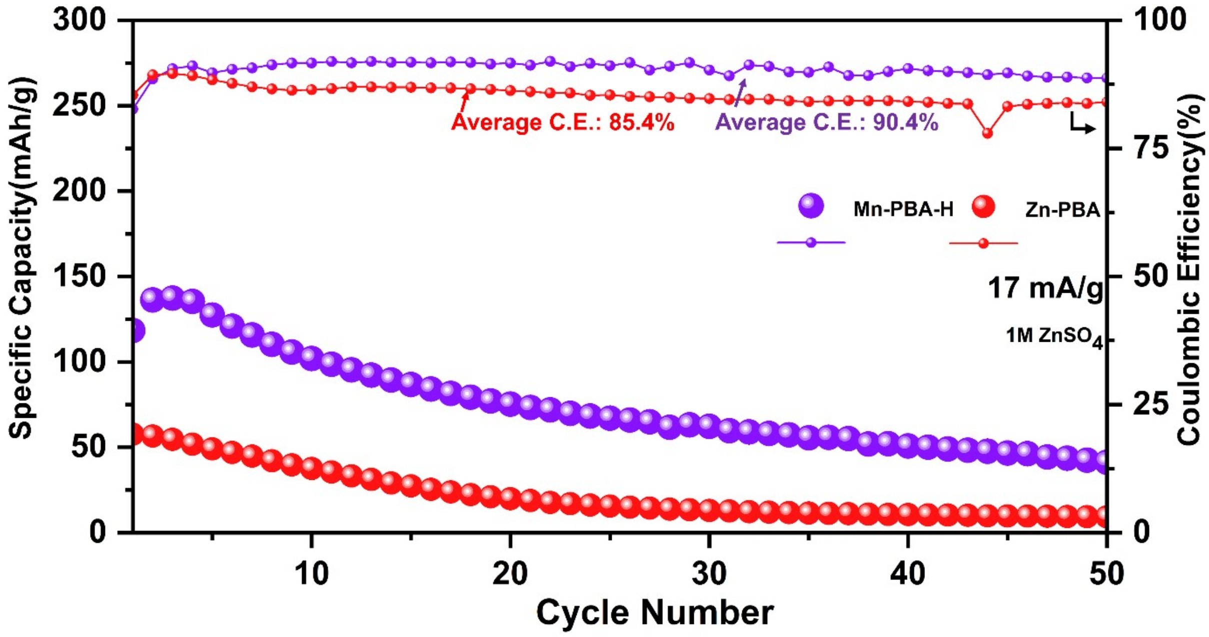

Figure S13. Long-term cycling stability of the Mn-PBA-H and Zn-PBA in 1M $ZnSO_4$ at 17 mA g^{-1}.

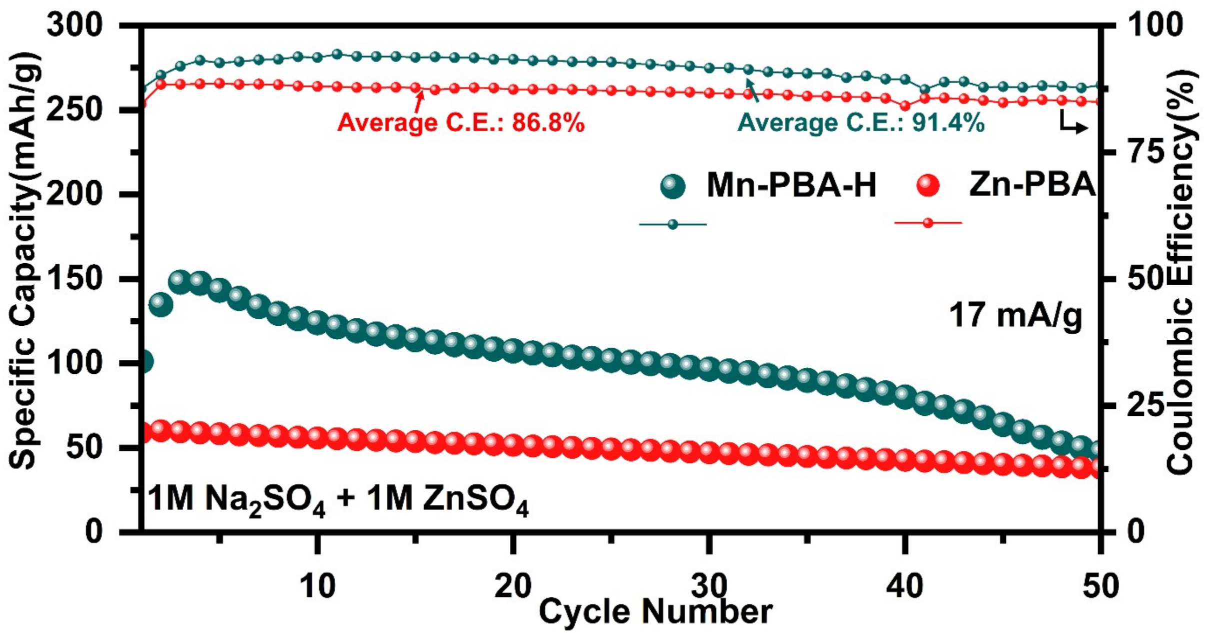

Figure S14. Long-term cycling stability of the Mn-PBA-H and Zn-PBA in 1M Na_2SO_4 + 1M $ZnSO_4$ at 17 mA g^{-1}.

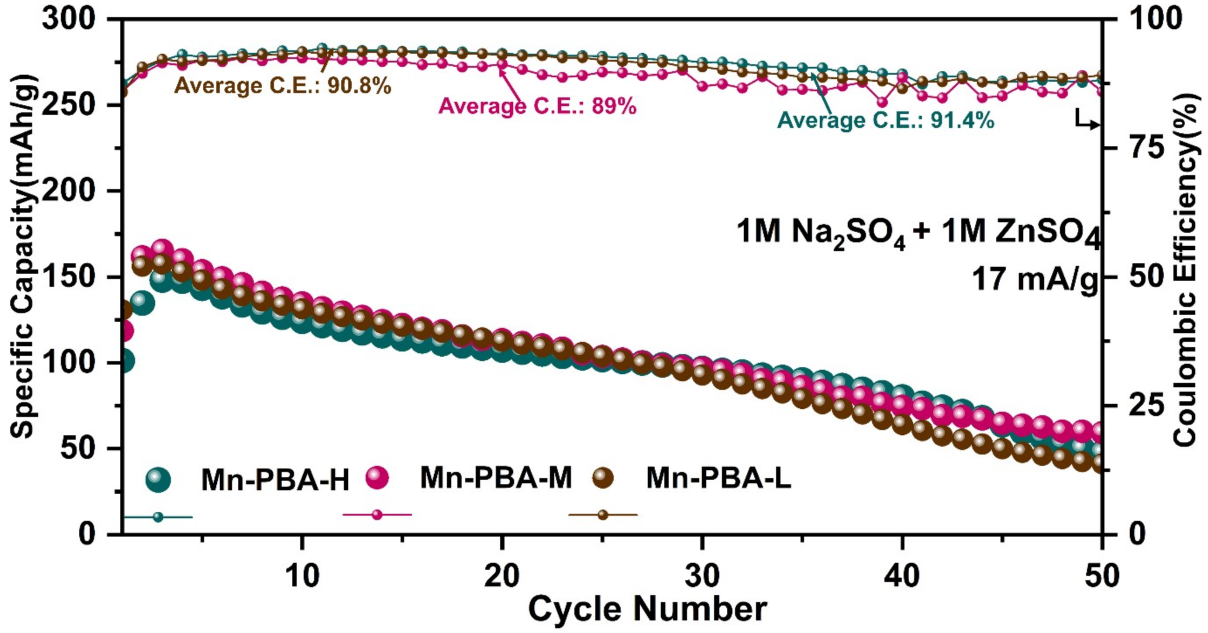

Figure S15. Long-term cycling stability of the all the variants in 1M Na_2SO_4 + 1M $ZnSO_4$ at 17 mA g^{-1}.

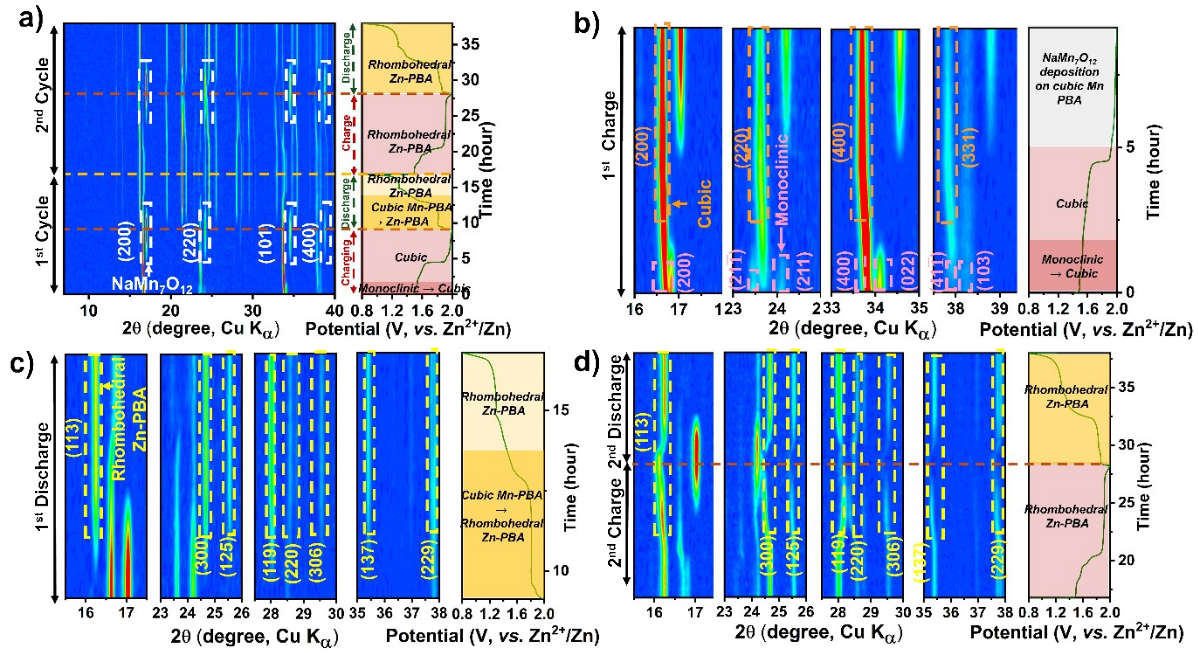

Figure S16. Operando XRD contour plots showing the phase evolution of Mn-PBA-M in 1 M Na_2SO_4 + 1 M $ZnSO_4$ electrolyte. (a) show the first two charge–discharge cycles, (b) the first charge, (c) the first discharge, and (d) the second charge–discharge cycle. Key phase transformations and structural evolutions during cycling are highlighted by arrows, labels, and legends.

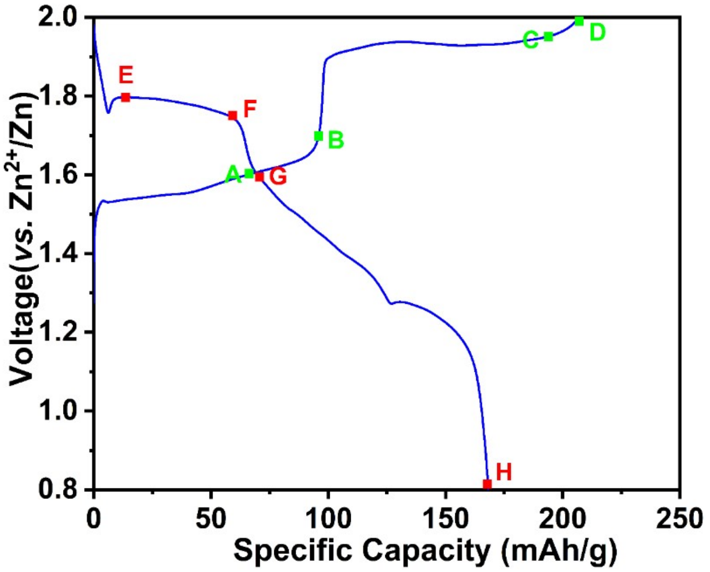

Figure S17. Voltage profile of Mn-PBA-H in 1M Na_2SO_4 + 1M $ZnSO_4$ at C/10 (17mA/g) with voltage points representing ex-situ XAS study

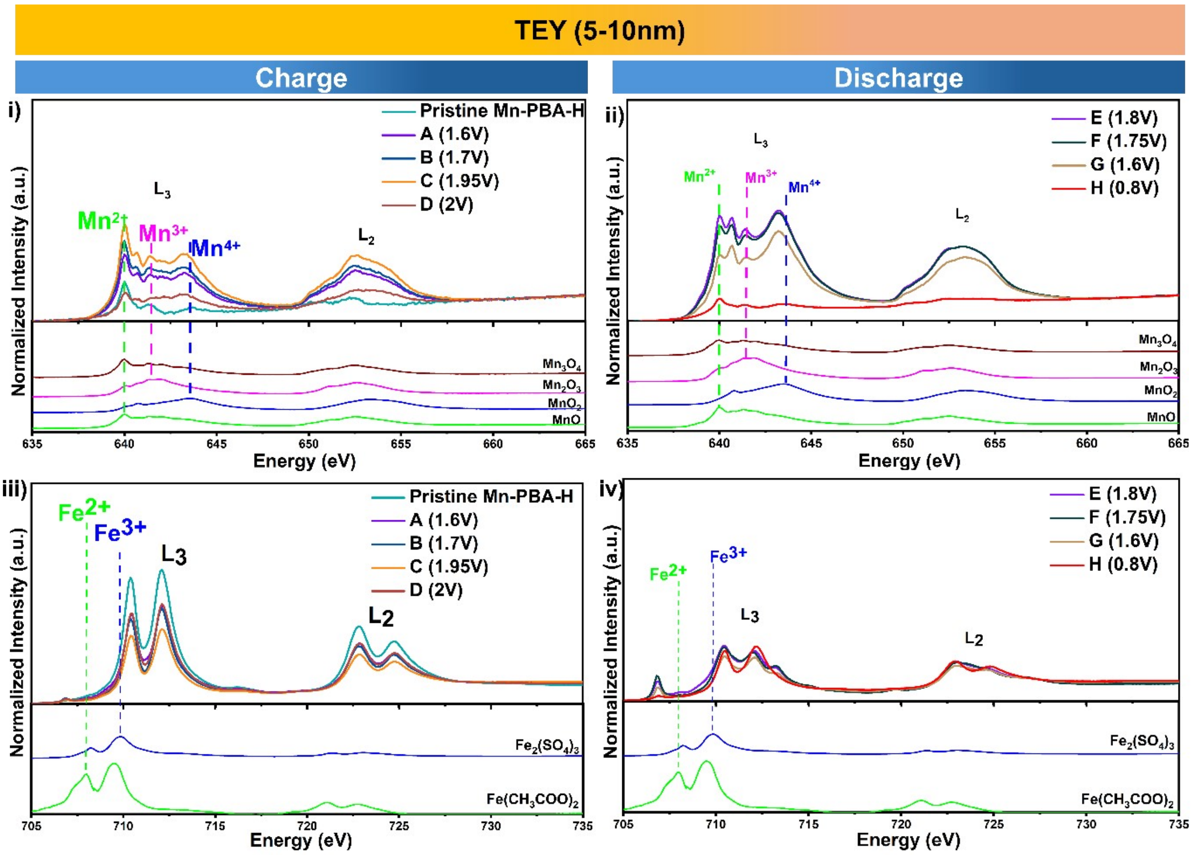

Figure S18. Surface sensitive XAS TEY L-edge of Mn and Fe during 1st charge/discharge.

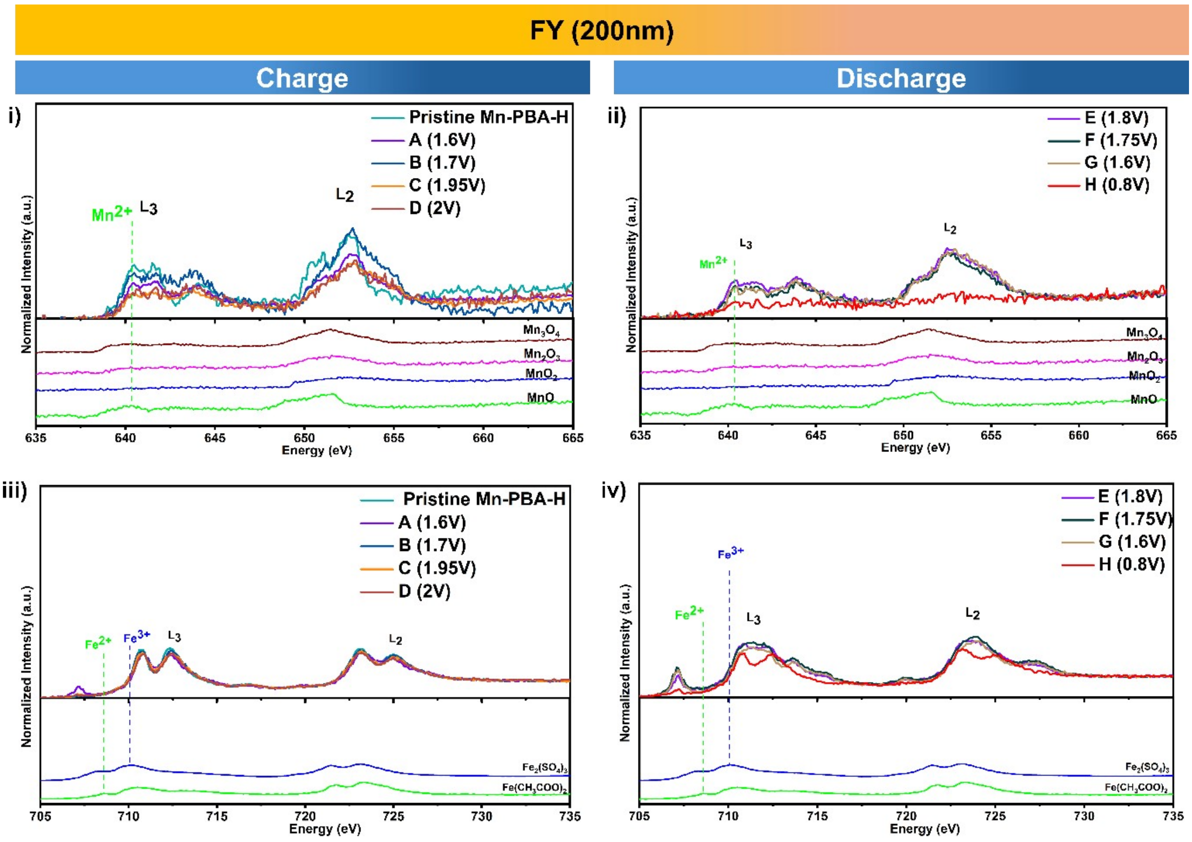

Figure S19 Surface sensitive XAS FY L-edge of Mn and Fe during 1st charge/discharge.

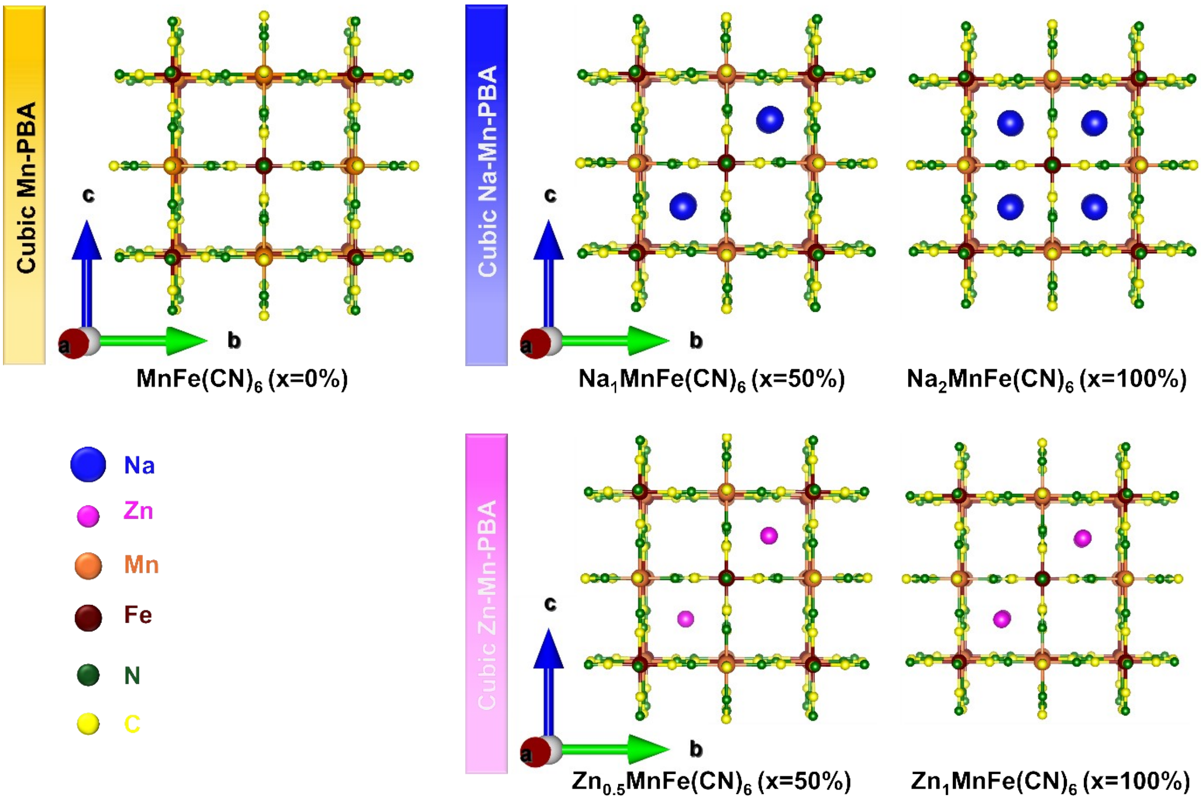

Figure 20. The relaxed structures showing the fully deintercalated Mn-PBA framework alongside the respective half-filled and full-filled Na^{+} and Zn^{2+} ions in the Mn-PBA framework

Table S2 Lattice parameters and the volume change in the simulated structures

Structures	Lattice Parameters						Volume (Å^3)	Volume Change (%)
	a (Å)	b (Å)	c (Å)	α (°)	β (°)	γ (°)		
Cubic Mn-PBA (x=0%)	10.65	10.65	10.65	90	90	90	1207.81	
Na-Mn-PBA (x=50%)	10.65	10.08	10.08	90	90	90	1083.12	-10.32
Na-Mn-PBA (x=100%)	10.13	10.13	10.13	90	90	90	1040.82	-13.83
Zn-Mn-PBA (x=50%)	10.46	10.46	10.46	90	90	90	1144.98	-5.20
Zn-Mn-PBA (x=100%)	10.28	10.60	10.60	90	90	90	1153.99	-4.46

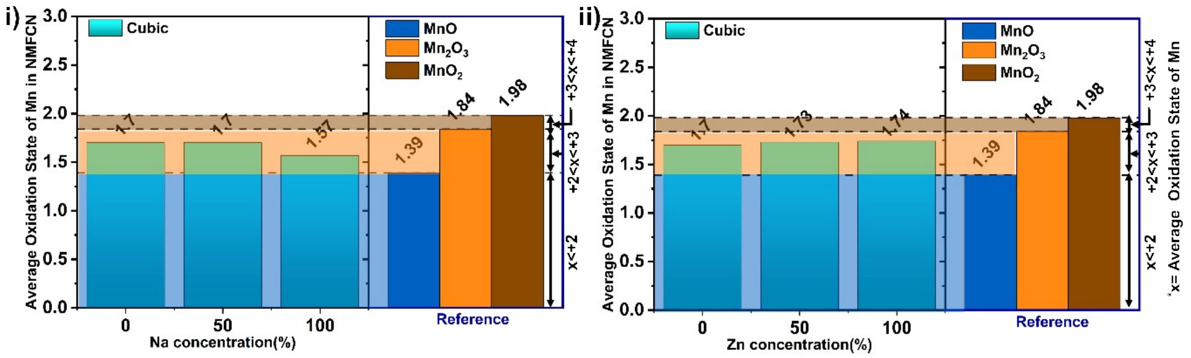

Figure 21. Average Mn oxidation state calculated from Bader charge analysis as a function of (**i**) Na^+ and (**ii**) Zn^{2+} intercalation in the Mn-PBA framework.
